# Virulence in Mice of a *Toxoplasma gondii* Type II Isolate Does Not Correlate With the Outcome of Experimental Infection in Pregnant Sheep

**DOI:** 10.3389/fcimb.2018.00436

**Published:** 2019-01-04

**Authors:** Roberto Sánchez-Sánchez, Ignacio Ferre, Javier Regidor-Cerrillo, Daniel Gutiérrez-Expósito, Luis Miguel Ferrer, Noive Arteche-Villasol, Javier Moreno-Gonzalo, Joachim Müller, Adriana Aguado-Martínez, Valentín Pérez, Andrew Hemphill, Luis Miguel Ortega-Mora, Julio Benavides

**Affiliations:** ^1^SALUVET, Animal Health Department, Faculty of Veterinary Sciences, Complutense University of Madrid, Madrid, Spain; ^2^Instituto de Ganadería de Montaña (CSIC-Universidad de León), León, Spain; ^3^Departamento de Patología Animal, Facultad de Veterinaria, University of Zaragoza, Zaragoza, Spain; ^4^Vetsuisse Faculty, Institute of Parasitology, University of Bern, Bern, Switzerland

**Keywords:** *Toxoplasma gondii*, type II, virulence, phenotypic traits, mice, sheep, congenital toxoplasmosis

## Abstract

*Toxoplasma gondii* is an apicomplexan parasite that infects almost all warm-blooded animals. Little is known about how the parasite virulence in mice extrapolates to other relevant hosts. In the current study, *in vitro* phenotype and *in vivo* behavior in mice and sheep of a type II *T. gondii* isolate (TgShSp1) were compared with the reference type II *T. gondii* isolate (TgME49). The results of *in vitro* assays and the intraperitoneal inoculation of tachyzoites in mice indicated an enhanced virulence for the laboratory isolate, TgME49, compared to the recently obtained TgShSp1 isolate. TgShSp1 proliferated at a slower rate and had delayed lysis plaque formation compared to TgME49, but it formed more cyst-like structures *in vitro*. No mortality was observed in adult mice after infection with 1–10^5^ tachyzoites intraperitoneally or with 25–2,000 oocysts orally of TgShSp1. In sheep orally challenged with oocysts, TgME49 infection resulted in sporadically higher rectal temperatures and higher parasite load in cotyledons from ewes that gave birth and brain tissues of the respective lambs, but no differences between these two isolates were found on fetal/lamb mortality or lesions and number of *T. gondii*-positive lambs. The congenital infection after challenge at mid-pregnancy with TgShSp1, measured as offspring mortality and vertical transmission, was different depending on the challenged host. In mice, mortality in 50% of the pups was observed when a dam was challenged with a high oocyst dose (500 TgShSp1 oocysts), whereas in sheep infected with the same dose of oocysts, mortality occurred in all fetuses. Likewise, mortality of 9 and 27% of the pups was observed in mice after infection with 100 and 25 TgShSp1 oocysts, respectively, while in sheep, infection with 50 and 10 TgShSp1 oocysts triggered mortality in 68 and 66% of the fetuses/lambs. Differences in vertical transmission in the surviving offspring were only found with the lower oocyst doses (100% after infection with 10 TgShSp1 oocysts in sheep and only 37% in mice after infection with 25 TgShSp1 oocysts). In conclusion, virulence in mice of *T. gondii* type II isolates may not be a good indicator to predict the outcome of infection in pregnant sheep.

## Introduction

*Toxoplasma gondii* is an apicomplexan parasite capable of infecting almost all warm-blooded animals and causing potentially fatal disease in humans and some relevant domestic species, such as small ruminants (Dubey, 2010). *Toxoplasma gondii* may be transmitted horizontally by oral ingestion of infectious oocysts from the environment and tissue cysts contained in raw and undercooked meat or vertically by transplacental transmission of tachyzoites (Tenter et al., 2000). *Toxoplasma gondii* diverges in three main clonal lineages, I, II, and III, with marked differences in mouse virulence (Howe and Sibley, 1995) although atypical strains have been also described (Su et al., 2006). Type I isolates are highly virulent in mice, whereas types II and III show a dose-dependent mortality (Saeij et al., 2006). Laboratory isolates from types I, II, and III are used in *T. gondii* research and have been maintained in successive passages in cell culture and mice. Enhanced virulence throughout successive passages in cell culture and mice for *T. gondii* type I isolates (e.g., RH isolate) has been widely reported (Villard et al., 1997; Mavin et al., 2004; Khan et al., 2009), but little is known about the influence of continuous passages in type II isolates. Likewise, there is little information on how *T. gondii* virulence in mice compares to virulence in other species, particularly in those experiencing clinical toxoplasmosis, such as sheep and humans.

Transmission of *T. gondii* from dams to offspring during pregnancy (congenital toxoplasmosis) is one of the consequences of infection (Innes et al., 2009; McAuley, 2014). Many animal experimental models have been developed; among them, mouse models are the most frequently used due to similarities in placental histology between rodents and humans (Vargas-Villavicencio et al., 2016). However, the structure of the placenta, reproductive physiology and immune responses greatly differ between rodents and ruminants, which clearly could influence the passing of *T. gondii* through the maternofetal interface and the modulation of the host immune responses during pregnancy (Entrican, 2002). In sheep, *T. gondii* is one of the main abortifacient agents (Dubey, 2009). Type II *T. gondii* isolates are the most prevalent in all of the hosts in Europe, including sheep (Chessa et al., 2014). In most of the experimental studies in pregnant sheep, type II isolates M1, M3, and M4 have been used (Dubey, 2009; Castaño et al., 2014), but the virulence in mice of these isolates was never assessed.

The aim of this study was to compare the phenotype *in vitro* and the virulence in mice of a newly obtained *T. gondii* type II isolate (TgShSp1) with that of the laboratory type II reference isolate (TgME49) and to compare congenital infection in mice and in sheep.

## Materials and Methods

### Ethics Statement

Animal procedures for the *T. gondii* isolation by a mouse bioassay of field samples from sheep abortions (PROEX 274/16), for evaluation of virulence by intraperitoneal inoculation of tachyzoites in non-pregnant mice (PROEX 274/16) and for *T. gondii* infection of mice and cats (PROEX 166/14) for oocyst production were approved by the Animal Welfare Committee of the Community of Madrid, Spain, following proceedings described in Spanish and EU legislation (Law 32/2007, R.D. 53/2013, and Council Directive 2010/63/EU). Animal procedures to characterize TgShSp1 oocysts in pregnant mice were approved by the Animal Welfare Committee of the Canton of Bern (approval No. BE 101/17). All sheep handling practices were approved by the local government and followed the recommendations of the Directive 2010/63/EU of the European Parliament, the Council on the protection of animals used for scientific purposes, and the IGM-CSIC Animal Experimentation Committee (protocol number 416-2016). All animals used in this study were handled in strict accordance with good clinical practices, and all efforts were made to minimize suffering.

### *T. gondii* Isolates, Isolation of the TgShSp1, and Genotyping

The *T. gondii* type II ovine isolate TgME49 (genotype #1) isolated in 1958 from sheep muscle (Lunde and Jacobs, 1983) was kindly donated by Dr. J. C. Boothroyd and had an unknown passage number, but it had been routinely maintained in cell culture and mice. The *T. gondii* type II (genotype #3) isolate (TgShSp1) was obtained from a *T. gondii* ovine abortion outbreak in September and October 2015 in a Spanish sheep flock (Assaf breed) in the province of Palencia (northwest Spain), which suffered abortion in 30 out of 239 pregnant sheep (12.5%).

TgShSp1 was isolated by passage in mice from a brain of an ovine aborted fetus, in which *T. gondii* infection was confirmed by PCR within 24 h after collection as described (Regidor-Cerrillo et al., 2008). Briefly, fetal brain (6 g) was homogenized in 6 mL of PBS containing 2% antibiotic-antimycotic solution (Gibco, Thermo Fisher Scientific, Waltham, MA, USA), filtered in sterile gauze, and centrifuged at 1,350 g for 15 min. The supernatant was discarded, and the sediment was suspended in 1,400 μL of PBS with antibiotic-antimycotic solution, and 400 μL was inoculated subcutaneously into one 8-weeks-old female CD1 mouse that was followed for clinical signs. At day 40 pi, asymptomatic mouse were euthanized, and the brain collected and tested by PCR (see below). The PCR-positive mouse brain was homogenized in 1,000 μL of PBS with antibiotics by passing through a descending series of needles (20–25 G) and immediately subcutaneously inoculated into two other CD1 mice (500 μL to each mice). At 11 dpi, mice were euthanized, and peritoneal flushes were used for isolation in MARC-145 cell culture (Regidor-Cerrillo et al., 2008) and checked for the presence of parasite by PCR. *Toxoplasma gondii* tachyzoites were observed in cell cultures at 4 days after inoculation of peritoneal flushes on MARC-145 cells and maintained by successive passages until cryopreservation.

The *T. gondii* isolate obtained was genotyped by polymerase chain reaction–restriction fragment length polymorphism (PCR-RFLP) using 12 molecular markers (SAG1, 3′-SAG2, 5′-SAG2, Alt.SAG2, SAG3, BTUB, GRA6, c22-8, c29-2, L358, PK1, and Apico) as previously described (Su et al., 2006). The digested PCR products were visualized by 2.5% agarose gel electrophoresis, stained with Gel Red® Nucleic Acid Gel Stain (Biotium®, Fremont, California, USA), observed under UV light, assigned to a *T. gondii* type and classified according to genotypes present in ToxoDB (http://toxodb.org/toxo/).

### *In vitro* Assays

MARC-145, Vero and Human Foreskin Fibroblasts (HFF) cell lines were used for studying the tachyzoite-to-bradyzoite conversion and proliferation of *T. gondii* isolates *in vitro*. Cells were routinely maintained in DMEM (Gibco, Thermo Fisher Scientific, Waltham, MA, USA) with phenol red supplemented with 10% heat-inactivated, sterile, filtered fetal calf serum (FCS) (Gibco, Thermo Fisher Scientific, Waltham, MA, USA), 2 mM glutamine (Lonza Group, Basel, Switzerland) and a mixture of penicillin (100 U/ml), streptomycin (100 μg/ml), and amphotericin B (Lonza Group, Basel, Switzerland) at 37°C in a humidified atmosphere of 5% CO_2_. TgME49 and TgShSp1 were maintained by serial passages in MARC-145 and Vero cells in the same culture medium with 2% FCS. Tachyzoites used for *in vitro* assays were harvested 3 days pi (TgME49) or 5 days pi (TgShSp1, passage 10), when the majority of parasites were still intracellular and purified by a PD-10 column (GE Healthcare, Little Chalfont, United Kingdom), as described (Regidor-Cerrillo et al., 2011). Tachyzoite viability was confirmed by trypan blue exclusion, and numbers were determined by counting in a Neubauer chamber. All assays were carried out in triplicate, including at least three replicates in each assay.

#### Evaluation of the Tachyzoite-to-Bradyzoite Differentiation *in vitro* Through Cyst Wall-Specific DBL Staining

Purified *T. gondii* parasites of the TgME49 or TgShSp1 isolate (2 × 10^3^ tachyzoites) were added to MARC-145 monolayers grown to confluence in 24-well plates. At 24 h, culture medium was replaced by DMEM with alkaline pH (8–8.2), and plates were incubated at 37°C without CO_2_ supplementation for 3–4 days (Skariah et al., 2010) to evaluate induced differentiation. Duplicate plates were maintained for 3 days under regular conditions to evaluate spontaneous differentiation (pH 7.2–7.4 and 5% CO_2_). Tachyzoite-to-bradyzoite conversion was evaluated at 72–96 h by double immunofluorescence staining in cell monolayers fixed with paraformaldehyde 3% and glutaraldehyde 0.05% and permeabilized with Triton X-100 0.25%, using a polyclonal mouse-anti *T. gondii* antiserum at a dilution of 1:100 as primary antibody, Alexa Fluor® 594 Goat Anti-Mouse IgG (H + L) (Life technologies, Carlsbad, CA, USA) at a dilution of 1:1,000 as secondary antibody for parasite staining, and the *Dolichos biflorus* lectin (DBL) (Vector Labs, Burlingame, United States) at a dilution of 1:50 for cyst wall staining. Cell nuclei were stained with DAPI. Finally, the total numbers of DBL-positive cysts and DBL-negative structures compatible with parasite structures, including lysis plaques, were counted using an inverted fluorescence microscope (Nikon Eclipse TE200) at 200x magnification. The percentage of conversion for each well was determined.

#### *In vitro* Intracellular Proliferation Assays

*Toxoplasma gondii* proliferation was evaluated in Vero cells by a plaque assay and in HFF determining the tachyzoite yield at 48 h pi. For this assay, Vero cultures grown to confluence in 24-well plates were infected with 5 × 10^4^ purified tachyzoites of either TgME49 or TgShSp1 and further maintained at 37°C and 5% CO_2_ for 4 days and stained with 0.2% crystal violet (Alfa Aesar, Haverhill, Massachusetts, United States) solution in 2% ethanol (Ufermann et al., 2017). Images were captured using a SMZ1000 binocular loupe (Nikon®, Tokyo, Japan).

Tachyzoite yield in HFF was determined by quantifying the number of tachyzoites at 48 h pi (TY_48h_) by real-time PCR (qPCR). HFF cultures grown to confluence in 24-well plates were infected with 10^5^ purified *T. gondii* tachyzoites and maintained for 48 h at 37°C in 5% CO_2_ as previously described (Regidor-Cerrillo et al., 2011). Then, the medium was removed, and cells were recovered in 150 μL lysis buffer and 10 μL proteinase K (Macherey-Nagel, Düren, Germany) for DNA extraction and quantification of parasite genomic DNA by qPCR.

### *In vivo* Experimental Infections

#### Virulence Assessment in Non-pregnant Mice Intraperitoneally Inoculated With TgShSp1 and TgME49 Tachyzoites

TgShSp1 and TgME49 virulence was determined in mice following recommendations for standardization described by Saraf et al. (2017). For the *in vivo* challenge, tachyzoites of TgShSp1 (passaged 10 times in cell culture) and TgME49 (unknown passage number) were recovered from Vero cultures when they were still largely intracellular (>80% of undisrupted parasitophorous vacuoles) (Regidor-Cerrillo et al., 2010), repeatedly passed through a 27-gauge needle at 4°C and filtered through a 5-μm polycarbonate filter (IpPORE®, IT4IP, Louvain-la-Neuve, Belgium) (Saraf et al., 2017). The tachyzoite viability was determined by Trypan blue exclusion and counted in a Neubauer chamber. Tachyzoite 10-fold serial dilutions were performed starting from 10^5^ to 1 tachyzoite(s) suspended in 200 μL of PBS, and each dilution was inoculated intraperitoneally into five 8-weeks-old female CD1 mice (Janvier-Labs, Laval, France) within 30 min of harvesting the parasites from cell culture. Five control female mice were inoculated with PBS. Mice were observed daily and clinical signs (morbidity) were scored according to the description made by Arranz-Solis et al. (2015a). Briefly, scores were classified as 0 (no alterations), 1 (ruffled coat), 2 (rounded back), 3 (noticeable loss of body condition/severe weight loss), or 4 (nervous signs such as activity decrease, hind limb paralysis, walking in circles, or head tilt). As a humane endpoint, mice exhibiting evident loss of body condition (score of 3) or nervous signs (score of 4) were culled to limit unnecessary suffering. Mice with clinical scores of 0, 1, and 2 were euthanized at 6 weeks p.i. Samples of blood were collected from mice for serology by IFAT and brain and lung for parasite detection by PCR.

#### Generation, Purification, and Sporulation of *T. gondii* Oocysts

Oocysts of the TgShSp1 isolate were obtained through oral infection of cats as previously described (Müller et al., 2017). Briefly, ten 8-weeks-old female CD1 mice (Janvier-Labs, Laval, France) were inoculated intraperitoneally with 10^5^ tachyzoites of TgShSp1 (passage 10). At 2 months post-inoculation, mice were euthanized, and the brains were collected. Two 12-weeks-old kittens free of *T. gondii* and other relevant feline pathogens (Isoquimen S.L., Barcelona, Spain) were fed a pool of 5 brains each. Feces were collected from kittens daily and examined to detect shedding of *T. gondii* oocysts. Unsporulated oocysts were harvested from feces and sporulated by resuspending in 2% H_2_SO_4_ for 4 days at room temperature. Sporulated oocysts were kept at 4°C until used. The same batch of sporulated oocysts of the TgME49 and TgShSp1 was used in mouse and sheep infections. Sporulated oocysts of TgME49 originated from the same batch as described earlier (Müller et al., 2017).

#### Assessment of TgShSp1 Oocyst Infection in Pregnant Mice

TgShSp1 was evaluated in pregnant mice out similarly as previously described for TgME49 (Müller et al., 2017). CD1 females (50 mice) and males (25 mice) were purchased from Charles River Laboratories (Sulzberg, Germany) at the age of 8 weeks and were maintained in a common room under conventional day/night cycle housing conditions. Females at 9 weeks of age were synchronized with respect to estrus and were distributed into cages, where two females and one male were housed together for 3 days (during which 3 females died). Subsequently, the female mice were orally infected by gavage with high doses of oocysts: 2,000 oocysts (group A, *n* = 9) and 500 oocysts (group B, *n* = 9), an intermediate dose of oocysts: 100 oocysts (group C, *n* = 10) and a low dose of oocysts: 25 oocysts (group D, *n* = 10) suspended in 100 μL of carboxymethyl cellulose solution (0.5% in water) at day 7 post-mating. The control group (group E, *n* = 9) received carboxymethyl cellulose solution alone. Pregnancy was confirmed 2 weeks post-mating by weighing, and pregnant mice were then allocated into single cages to give birth on days 19–22 and to rear their pups for an additional 4 weeks. During this time, those females that had remained non-pregnant were maintained in cages of three to five mice. Dams and their offspring were evaluated daily from birth to day 28 post-partum (pp). Despite the numerous parameters evaluated, pup mortality (number of pups born dead or euthanized due to severe clinical signs as described above for intraperitoneal inoculation) and vertical transmission (surviving pups being PCR-positive in the brain) were the most relevant assessments. Data on pregnancy rate (percentage of female mice that became pregnant), litter size (number of delivered pups per dam), and clinical signs (morbidity) of dams and non-pregnant mice were recorded during this time as described above for intraperitoneal inoculation. Neonates were weighed every second day from day 14 pp until the end of the experiment (day 28 pp) to evaluate morbidity in the offspring. Day 14 pp was chosen as a starting point for weight monitoring to avoid excessive handling of the pups during the first 2 weeks after birth, which can result in rejection by the dams. Dams, non-pregnant mice and pups were euthanized in a CO_2_ chamber at 28 days pp. Blood from dams and non-pregnant mice was recovered by cardiac puncture, and sera were obtained to test humoral immune responses. Brains and lungs were removed from dams and non-pregnant mice and stored at −20°C until determination of parasite load. The heads of pups that survived were collected and stored at −20°C. Subsequently, the frozen heads were cleaved and brains were removed. The frozen brains from pups were immediately processed for DNA purification and then parasite quantification. Whenever possible, dead pups succumbing to the infection early after birth were removed, their heads sampled and their brains analyzed.

#### Assessment of TgShSp1 and TgME49 Oocyst Infections in Pregnant Sheep

Fifty-four pure Rasa Aragonesa breed female ewes aged 12 months were selected from a commercial flock. All animals were seronegative for *T. gondii, N. caninum*, border disease virus (BDV), Schmallenberg virus (SBV), *Coxiella burnetii*, and *Chlamydia abortus* as determined by enzyme-linked immunosorbent assay (ELISA). They were estrus-synchronized and mated with pure-bred Rasa Aragonesa tups for 2 days, after which the rams were separated from the ewes. Pregnancy and fetal viability were confirmed by ultrasound scanning (US) on day 40 post-mating. Pregnant ewes (*n* = 37) were randomly distributed into seven experimental groups and housed at the Instituto de Ganadería de Montaña (CSIC-Universidad de León), León, Spain.

Thirty-three pregnant ewes were orally dosed on day 90 of pregnancy with a high dose of oocysts (500 sporulated oocysts) of TgShSp1 (group 500A, G500A, *n* = 6) or TgME49 (group 500B, G500B, *n* = 5), an intermediate dose of oocysts (50 oocysts) of TgShSp1 (group 50A, G50A, *n* = 6) or TgME49 (group 50B, G50B, *n* = 5) or a low dose of oocysts (10 oocysts) of TgShSp1 (group 10A, G10A, *n* = 6) or TgME49 (group 10B, G10B, *n* = 5). The four remaining sheep were used as negative controls of infection (uninfected) and received 50 mL of PBS on day 90 of pregnancy.

Pregnant ewes were observed daily throughout the experimental period. Rectal temperatures were recorded daily from day 0 until 14 days pi and then weekly to evaluate morbidity. The physiological range for rectal temperatures in sheep was obtained from Diffay et al. (2002), and rectal temperatures above 40°C were considered hyperthermic. Fetal viability was assessed by US monitoring of fetal heartbeat and movements twice a week after infection. When fetal death occurred, or immediately after parturition, dams and lambs were first sedated with xylazine (Rompun, Bayer, Mannheim, Germany) and then euthanized by an intravenous overdose of embutramide and mebezonium iodide (T61, Intervet, Salamanca, Spain). Animals from the uninfected group were examined by US every 2 weeks.

According to the survival in fetuses/lambs, sheep were classified into three categories: (a) suffering early abortions (i.e., between 8 and 11 dpi); (b) suffering late abortions, which occurred from 12 to 50 dpi; and (c) sheep delivering stillbirths, mummified fetuses or live lambs from 51 dpi. After birth, lambs were clinically inspected and then sedated and euthanized. Lambs showing weakness in relation to all live lambs were used to calculate morbidity in the offspring. In spite of the numerous parameters evaluated, fetal/lamb mortality and vertical transmission in live lambs (seropositivity and parasite detection in brain or lung) were the most relevant assessments.

Blood samples to evaluate humoral immune responses were collected prior to infection, at 3, 5, 7, and 10 days pi and then weekly by jugular blood draw. Precolostral serum was collected from lambs immediately after delivery from dams. To prevent any transmission of colostral antibodies from dams, udders were covered with a piece of cloth 1 week before the expected date of delivery as a preventive measure, and lambs were separated from their mothers immediately after birth. Serum samples were stored at −80°C until analysis.

During necropsy, six randomly selected placentomes or cotyledons from aborted dams and dams that gave birth, respectively, were recovered from each placenta, transversally cut into 2–3 mm-thick slices, and fixed in 10% formalin for histopathological examination, whereas the remaining tissues from these placentomes/cotyledons were stored at −80°C for further DNA extraction and PCR analyses. Samples from fetal tissues, including brain and lungs, were stored at −80°C for DNA extraction or were fixed in 10% formalin for histopathology. Thoracic and abdominal fluids were also collected from fetuses and stillborn lambs from which precolostral sera could not be obtained, and maintained at −80°C for serology.

### Serological Analyses: IFAT and ELISA

The serum samples from mice used for isolation and determination of virulence were analyzed by the immunofluorescence antibody test (IFAT) for the detection of anti-*T. gondii* IgG as previously described (Alvarez-Garcia et al., 2003), using an anti-mouse IgG conjugated to FITC (Sigma-Aldrich, Madrid, Spain) diluted 1:64 in Evans Blue (Sigma-Aldrich). We used the cut-off of 1:25. Serum titers for *T. gondii* in oocyst-infected mice were assessed by ELISA as previously described for *Neospora caninum*-infected mice (Debache et al., 2008, 2009), except that soluble antigen extract from *T. gondii* tachyzoites was used (Alaeddine et al., 2005).

*Toxoplasma gondii*-specific IgG antibody levels in sheep were measured using an in-house indirect ELISA similarly as previously described (Castaño et al., 2014). The indirect fluorescent antibody test (IFAT) was used to detect specific IgG anti-*Toxoplasma* antibodies in fetal fluids and precolostral sera, adapting the technique previously described for IFAT analysis in *N. caninum*-infected animals (Alvarez-Garcia et al., 2003), using an anti-sheep IgG (Sigma-Aldrich) diluted 1:200 in Evans blue (Sigma-Aldrich). Fetal fluids and precolostral sera were diluted at 2-fold serial dilutions in PBS starting at 1:8 (for fetal fluids) and 1:50 (for precolostral sera) up to the endpoint titer. Continuous tachyzoite membrane fluorescence at a titer ≥8 for fetal fluids or ≥50 for precolostral sera was considered a positive reaction.

### DNA Extraction and PCR for Parasite Detection and Quantification in Tissues

Genomic DNA from *in vitro* samples was extracted from these samples using the NucleoSpin® DNA RapidLyse Kit (Macherey-Nagel, Düren, Germany) according to the manufacturer's instructions. DNA concentrations were adjusted to 20 ng/μL and quantified using qPCR with primer pairs for the 529-bp repeat element for *T. gondii* for parasite quantification and primer pairs for the 28S rRNA gene for quantify cell DNA under conditions previously described (Collantes-Fernández et al., 2002; Castaño et al., 2016, respectively).

Genomic DNA was extracted from mice that were used for isolation and determination of virulence out using the commercial Maxwell® 16 Mouse Tail DNA Purification Kit. The *T. gondii* DNA detection was carried out by an ITS-1 PCR adapted to a single tube as previously described (Castaño et al., 2014). DNA extraction and qPCR analysis from oocyst-infected mice were performed as previously described (Müller et al., 2017).

In sheep, genomic DNA was extracted from three 50–100 mg samples taken from each location: six placentomes in aborted dams or six cotyledons in dams that gave birth, as well as fetal brain and lung, using the commercial Maxwell® 16 Mouse Tail DNA Purification Kit. *T. gondii* DNA detection was carried out by an ITS-1 PCR as described above (Castaño et al., 2014). DNA that tested positive by nested-PCR was adjusted to 20 ng/μL and quantified using qPCR as previously described (Castaño et al., 2014). Parasite number in tissue samples (parasite burden) was expressed as parasite number/mg ovine tissue. Standard curves for *T. gondii* and sheep DNA showed an average slope of −3.44 and −3.30, respectively, and an *R*^2^ > 0.99. Parasite-negative DNA samples were included in each round of DNA extraction and PCR as negative controls.

### Histological Processing

After fixation for 5 days, placental and fetal sheep tissues were cut coronally, embedded in paraffin wax and processed by standard procedures for hematoxylin and eosin (HE) staining. Conventional histological evaluation was carried out on all sections. To quantify the lesions in the brain of stillborn lambs and live lambs, the number and size of glial foci, as well as the total area of lesion in the examined tissue, were calculated through a computer-assisted morphometric analysis on HE-stained sections following the procedure described previously (Arranz-Solis et al., 2015b).

### Statistical Analysis

The growth rate and percentage of DBL-positive cysts *in vitro* of TgME49 and TgShSp1 were compared using the Mann–Whitney test. In pregnant mice, differences in seroconversion, pregnancy rates, litter size, pup mortality, and parasite presence in tissues were analyzed by the χ^2^-test or Fisher's exact *F*-test. One-way ANOVA followed by Tukey's multiple comparisons test were employed to compare body weights. Parasite burdens and anti-*T. gondii* antibody levels were analyzed using the non-parametric Kruskal–Wallis test followed by Dunn's test for comparisons between groups, as well as the Mann–Whitney test for pairwise comparisons.

In pregnant sheep, the number of fetuses/lambs suffering mortality and the number of weak lambs (morbidity) were compared using the χ^2^-test or Fisher's exact *F*-test. Rectal temperatures and humoral immune responses were analyzed using one-way ANOVA followed by Tukey's multiple comparisons test until 14 days pi or until the end of the experiment. Differences in frequency of PCR detection of parasite DNA and in the percentage of cases showing lesions were evaluated using the χ^2^-test or Fisher's exact *F*-test. Differences in parasite burdens and histological measurements of lesions were analyzed using the non-parametric Kruskal–Wallis test followed by Dunn's test for comparisons between groups, as well as the Mann–Whitney test for pairwise comparisons.

Differences between mice and sheep in the number of fetuses/pups/lambs that died in relation to the total number of fetuses/pups/lambs or in the number of surviving offspring infected with *T. gondii* in relation to all live offspring were assessed using the χ^2^-test or Fisher's exact F-test. Likewise, a categorization of the parameters to evaluate congenital infection was done, into high (>67%), medium (66–34%), low (< 33%), or none (0%) of the fetuses/pups/lambs with clinical signs, offspring mortality or vertical transmission. Statistical significance for all analyses was established at *P* < 0.05. All statistical analyses were performed using GraphPad Prism 6.01 software (San Diego, CA, USA).

## Results

### Isolation of the *T. gondii* Isolate TgShSp1 From a Sheep Flock in Spain

On day 40 post-inoculation, one mouse inoculated with the brain homogenate of the sheep abortion was *T. gondii* PCR-positive in the brain. The peritoneal fluid of one of the other two mice inoculated with the positive mouse brain was PCR-positive on day 11 pi. Four days after inoculating the PCR-positive peritoneal flush into cell culture, the isolation of TgShSp1 was confirmed. Genotyping classified TgShSp1 as genotype #3 (a type II variant, type II for nine alleles/type I for Apico) (ToxoDB).

### TgShSp1 and TgME49 Differ in Behavior *in vitro*

#### TgShSp1 Exhibits a High Capacity to Form Cysts *in vitro*

**Figure 1:**
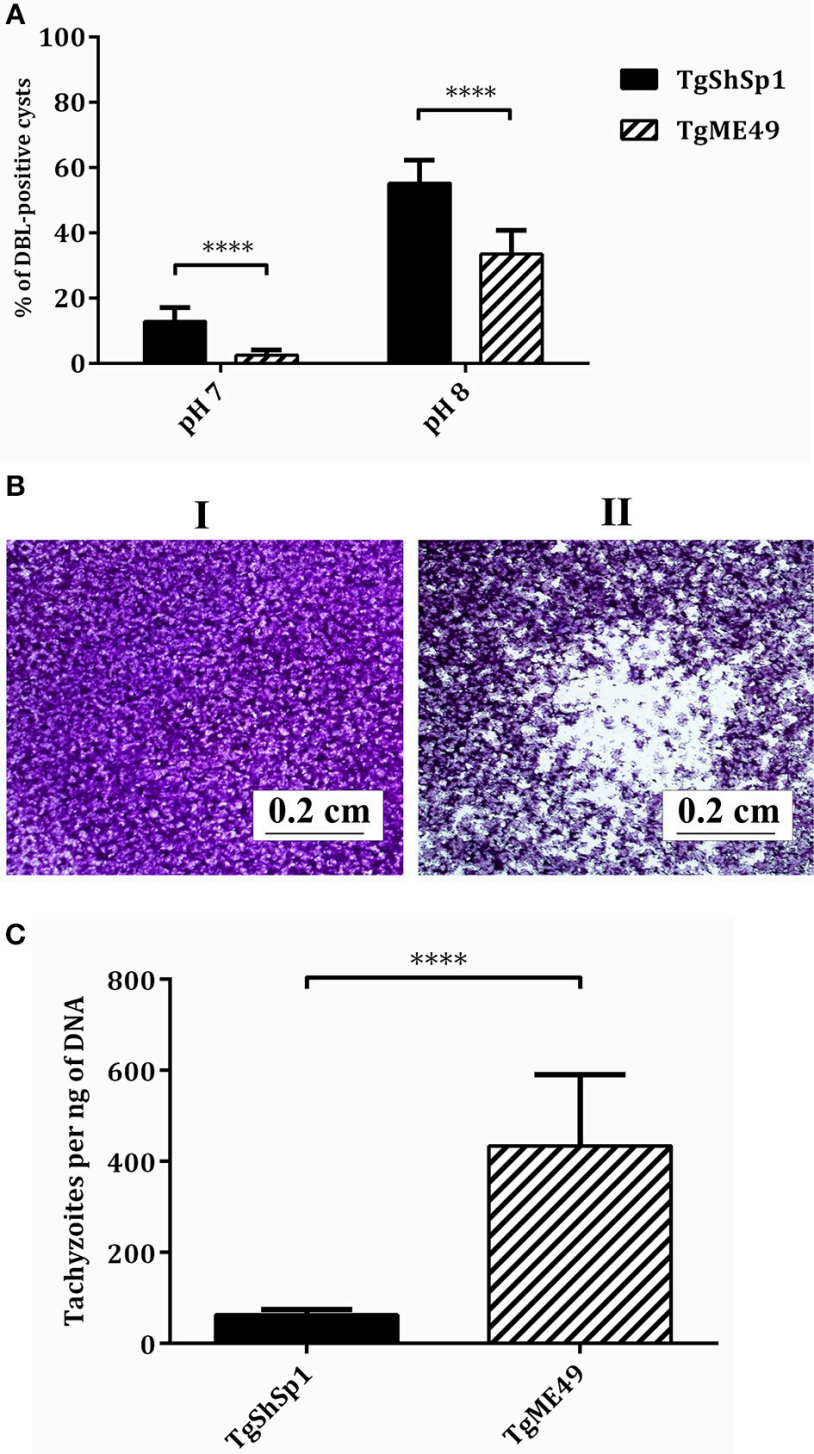
Tachyzoite-to-bradyzoite differentiation, plaque formation and tachyzoite yield *in vitro* of TgME49 and TgShSp1 isolates. **(A)** *In vitro* tachyzoite-to-bradyzoite differentiation at neutral and alkaline pH. Percentage of events being DBL-positive cysts at pH 7 and pH 8 are represented. The rest of non-represented events were found to be DBL-negative structures and lysis plaques. ^****^Marks the higher spontaneous and induced differentiation into bradyzoites with the TgShSp1 isolate. **(B)** Plaque formation with TgShSp1 (I) and TgME49 (II) isolates. Plaques are visible as clear zones on a crystal violet-stained Vero monolayer background. **(C)** A column-plot graph representing the tachyzoite yield (TY_48h_) of TgME49 and TgShSp1. Values of replicates from experiments performed in triplicate for each isolate. Error bars indicate the SD. ^****^Marks the significantly higher TY_48h_ values for TgME49 compared to TgShSp1.

At difference in TgME49 free-floating cyst-like structures was often identified by light microscopy in the TgShSp1 infected cultures at 3 days p.i. in successive passages. TgShSp1 cultured under regular conditions (at a neutral pH) demonstrated spontaneous conversion to bradyzoite with a statistically higher number of DBL-positive cysts (14%) compared to TgME49 (2%) (*P* < 0.0001). Additionally, after induction of bradyzoite development (at a basic pH), TgShSp1 formed a higher number of DBL-positive cysts (55%) compared to TgME49 (33%) (*P* < 0.0001) (Figure 1A).

#### TgME49 Shows a Higher Growth Rate *in vitro* Than TgShSp1

Evaluation of parasite growth in Vero cells by a lysis plaque assay showed that TgME49 produced large clear zones due to host cell lysis, while during the same period, TgShSp1 showed essentially an intact monolayer cell (Figure 1B). Determination of the TY_48h_ in HFF was also assessed to confirm differences in parasite growth. The TY_48h_ values for TgME49 were significantly higher compared to those from TgShSp1 (*P* < 0.0001) (Figure 1C).

### TgShSp1 and TgME49 Differ Greatly in Virulence in Mice

**Figure 2:**
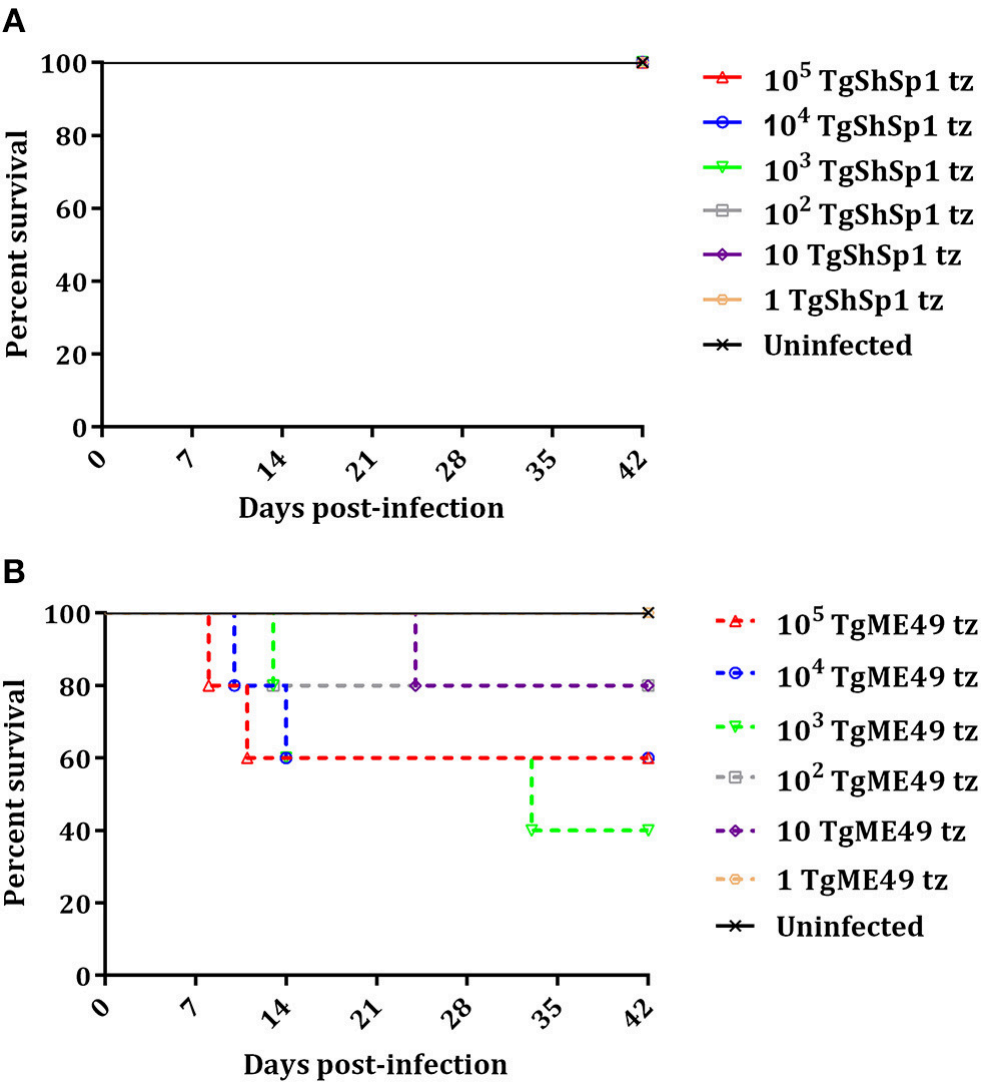
Survival curve of CD1 mice after infection with *T. gondii* tachyzoites of TgShSp1 isolate **(A)** or TgME49 isolate **(B)**. Five mice per group were infected i.p. with 10^5^, 10^4^, 10^3^, 10^2^, 10, or 1 tachyzoite of the TgShSp1 isolate or the TgME49 isolate. Survival was monitored for 42 days. Each point represents the percentage of surviving animals at that day, and downward steps correspond to euthanasia due to severe clinical signs.

A summary of clinical signs, serology and parasite detection in mice is shown in Table S1. Most of the mice inoculated with doses from 10^5^ to 10^2^ tachyzoites of TgShSp1 only exhibited a ruffled coat between days 4 and 13 pi, but they did not have to be euthanized due to severe clinical signs (Figure 2A; Table S1). In contrast, upon infection with TgME49, several mice had to be euthanized due to clinical scores (Figure 2B). The surviving mice infected with doses from 10^5^ to 10 tachyzoites of TgME49 exhibited clinical signs (rounded back) between days 8 and 14 pi (Table S1). The LD_50_ for TgME49 was approximately 10^3^ tachyzoites vs. >10^5^ tachyzoites for TgShSp1.

All mice inoculated with doses from 10^5^ to 10 tachyzoites of TgShSp1 were seropositive at 6 weeks pi, with IFAT titers ranging from 1:400 to 1:3,200. Concerning mice infected with TgME49, mice euthanized prior to day 24 pi were seronegative, while one mouse infected with 10^3^ tachyzoites euthanized on day 33 pi had an IFAT titer of 1:25. Survivors infected with doses from 10^5^ to 10 tachyzoites of TgME49 were seropositive at 6 weeks pi, with IFAT titers ranging from 1:50 to 1:800 (Table S1).

All mice infected with doses from 10^5^ to 10 tachyzoites of TgME49 and TgShSp1 were PCR-positive in the brain, confirming *T. gondii* infection. Likewise, almost all lung samples from mice infected with doses from 10^5^ to 10 tachyzoites of both isolates were PCR-positive, except lung samples from surviving mice infected with 10 tachyzoites of TgME49. Mice infected with 1 tachyzoite did not show clinical signs, they were seronegative, and all tissue samples were PCR-negative, identical to uninfected mice (Table S1).

### TgShSp1 Shows Low Virulence in Mice Infected With Oocysts but Is Efficiently Transmitted to Offspring

#### Evaluation of TgShSp1 Infection in Dams

Clinical signs in infected dams were generally mild. Therefore, none of the dams had to be euthanized due to severe clinical signs. At 12 days pi, one out of five dams infected with 2000 oocysts (group A) exhibited ruffled coat (1), and one out of six dams infected with 500 oocysts (group B) displayed rounded back (2). No clinical signs were observed in dams infected with 100, 25, or 0 oocysts (groups C, D, and E). Pregnancy rates ranged from 55 to 66%, with no significant differences between them. Similarly, no differences between the groups were found in litter size (11.8–14.4 delivered pups), suggesting that pregnancy was not noticeably altered by infection with TgShSp1 oocysts (Table 1).

**Table 1:** Effects of oral infection with TgShSp1 oocysts on infection status in adult mice, fertility in dams, mortality in pups, and vertical transmission in surviving offspring.

				**Non-pregnant mice**			**Pregnant mice**			**Pups**				
	**Oocysts**	***N*^**°**^**	**Pregnant^a^**	**Lung positive**	**Brain positive**	**Serum positive**	**Lung positive**	**Brain positive**	**Serum positive**	**Litter size (0 days *post-partum*)^b^**	**Mortality per litter (28 days *post-partum*)^c^**	**Litter with 100% mortality (28 days *post-partum*)^d^**	**Surviving (28 days *post-partum*)^e^**	**Brain positive (28 days *post-partum*)**
Group A	2,000	9	5/9	0/4	4/4	4/4	3/5	5/5	5/5	62	5/5	1/5	31 (50%)^*^	31 (100%)
Group B	500	9	6/9	0/3	3/3	3/3	5/6	6/6	6/6	71	5/6	2/6	36 (50%)^*^	36 (100%)
Group C	100	10	6/10	3/4	4/4	4/4	5/6	6/6	6/6	83	1/6	0/6	76 (91%)	76 (100%)
Group D	25	10	6/10	0/4	3/4	3/4	3/6	3/6	3/6	72	5/6	1/6	53 (73%)	20 (37%)^****^
Group E	Control	9	5/9							72	1/5	0/5	71 (98%)	

a *Number of pregnant mice/mice housed with males*.

b *Number of full-term delivered pups*.

c *Number of litters with at least one pup born dead or euthanized due to severe clinical signs/total number of litters*.

d *Number of litters in which all delivered pups born dead or have to be euthanized due to severe clinical signs/total number of litters*.

e *Number of pups surviving at day 28 pp (percentage)*.

* P < 0.05 and

**** *P < 0.0001 significant differences*.

All infected dams with 2,000, 500, and 100 oocysts (groups A, B, and C) developed *Toxoplasma*-specific humoral immune responses at day 28 pp. However, although only seroconversion in half of the dams was observed in the group infected with 25 oocysts (group D), there were no statistically significant differences in the number of dams showing seroconversion between infected groups (Table 1). Anti-*T. gondii* IgG levels were significantly increased in groups infected with 2,000 (*P* < 0.05), 500 (*P* < 0.01), and 100 oocysts (*P* < 0.01) in comparison to the unchallenged group in which all dams were seronegative (Figure S1A). *Toxoplasma gondii* DNA was detected in the brain of all dams from infected groups, with the exception of three dams in the group infected with 25 oocysts (group D) (Table 1). Quantitative evaluation of parasite burdens in brain showed no significant differences between infected groups (Figure S2A). In the lungs, parasite DNA was detected in the 50–83% of the dams from oocyst-infected groups, without significant differences in parasite detection or parasite load between them (Figure S3A; Table 1).

#### Evaluation of TgShSp1 Infection in Offspring Mice

**Figure 3:**
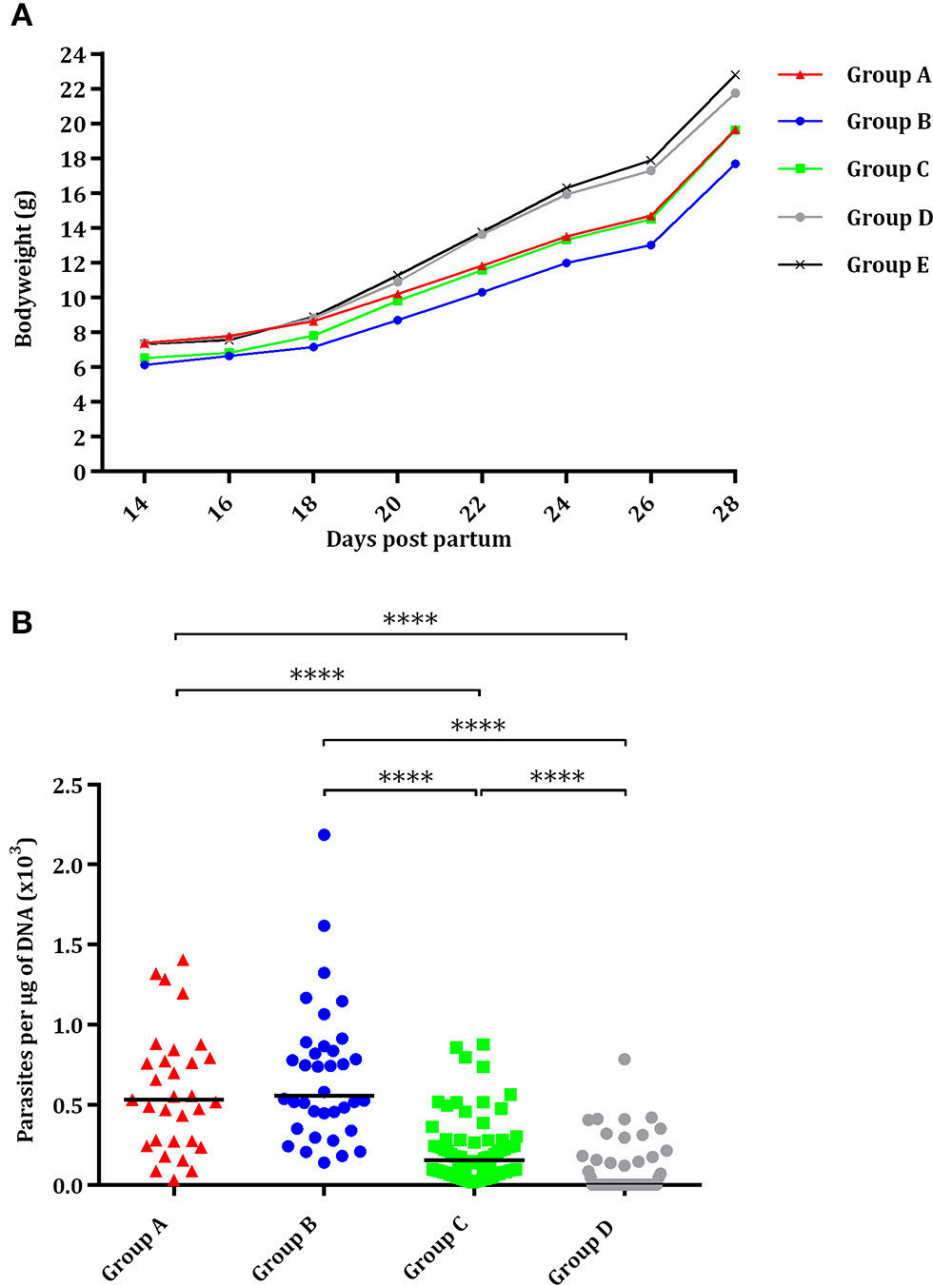
Effect of oral administration of TgShSp1 oocysts in the mouse pups. **(A)** Body weight progression of neonates born from dams infected on day 7 of pregnancy with 2,000 (group A), 500 (group B), 100 (group C), or 25 (group D) TgShSp1 oocysts and the uninfected group (group E). Each point represents the average body weight of all animals per group. **(B)** Dot-plot graph of *T. gondii* load in brain from surviving pups. Each dot represents individual values of parasite burden (number of parasites per μg of DNA), and medians are represented as horizontal lines. ^****^P < 0.0001.

Most pups were born dead (82/93; 88%), although a few pups had to be euthanized due to severe clinical signs between day 2 and day 21 pp (11/93; 12%). Half of the pups in the group infected with 2,000 oocysts (group A) and with 500 oocysts (group B) were born dead or had to be euthanized due to severe clinical signs and had significantly higher pup mortality compared to the uninfected group (*P* < 0.05) (Table 1). In groups infected with 100 oocysts (group C) and 25 oocysts (group D), respectively, 9 and 27% of pups were born dead or had to be euthanized due to severe clinical signs. Only one of seventy-two pups was born dead in the uninfected group (group E) (Table 1). Starting from day 14 pp, offspring of the uninfected group (group E) showed significantly higher body weight than groups infected with 500 (*P* < 0.0001) and 100 oocysts (*P* < 0.001), and the same was true of those infected with 2,000 oocysts (group A) from day 22 pp (*P* < 0.001). However, no decreased body weight was noted in pups infected with 25 oocysts (group D) (Figure 3A).

Vertical transmission was detected upon PCR analyses in all brains (100%) of surviving pups from groups infected with 2,000, 500, and 100 oocysts (groups A, B, and C). However, parasite was only detected in 37% of the brains from pups infected with 25 oocysts (group D), with significantly lower parasite detection compared to groups infected with 2,000, 500, and 100 oocysts (*P* < 0.0001) (Table 1). Pups infected with 25 oocysts (group D) and with 100 oocysts showed lower parasite burden compared to those infected with 2,000 and 500 oocysts (groups A and B) (*P* < 0.0001) (Figure 3B). No *T. gondii* DNA could be detected in the brain of pups that had died on day 0 or 1 pp.

#### Evaluation of TgShSp1 Oocyst Infection in Non-pregnant Mice

Similarly, to pregnant mice, clinical signs in non-pregnant mice infected with oocysts were generally mild. Therefore, none of the non-pregnant mice had to be euthanized due to severe clinical signs. Only ruffled coat was observed in all mice infected with 2,000 oocysts (group A), in one out of three mice in the group infected with 500 oocysts (group B) and in one out of four non-pregnant mice in the group infected with 100 oocysts (group C). All infected non-pregnant mice infected with 2,000, 500, and 100 oocysts (groups A, B, and C) developed *Toxoplasma*-specific humoral immune responses at day 28 p.p. that were significantly increased in comparison to the unchallenged group, with basal IgG levels (*P* < 0.05) (Figure S1B). In the group infected with 25 oocysts (group D), only three of the four non-pregnant mice seroconverted (Table 1). Further analyses of antibody responses of pregnant and non-pregnant mice in each group did not reveal any significant differences. *Toxoplasma gondii* DNA was detected in the brains of all non-pregnant mice from infected groups, with the exception of one mouse infected with 25 oocysts (group D) (Table 1). In the lungs, parasite DNA was only detected in three out of four samples from mice infected with 100 oocysts (group C). Quantitative evaluation showed no significant differences in brain and lung parasite loads in non-pregnant mice (Figures S2B, S3B). The comparison of parasite load in brain and lungs in pregnant mice vs. non-pregnant mice revealed no significant differences.

### TgShSp1 and TgME49 Oocyst Infection Cause Similar Fetal/Lamb Mortality and Vertical Transmission in Pregnant Sheep

#### Clinical Observations

**Figure 4:**
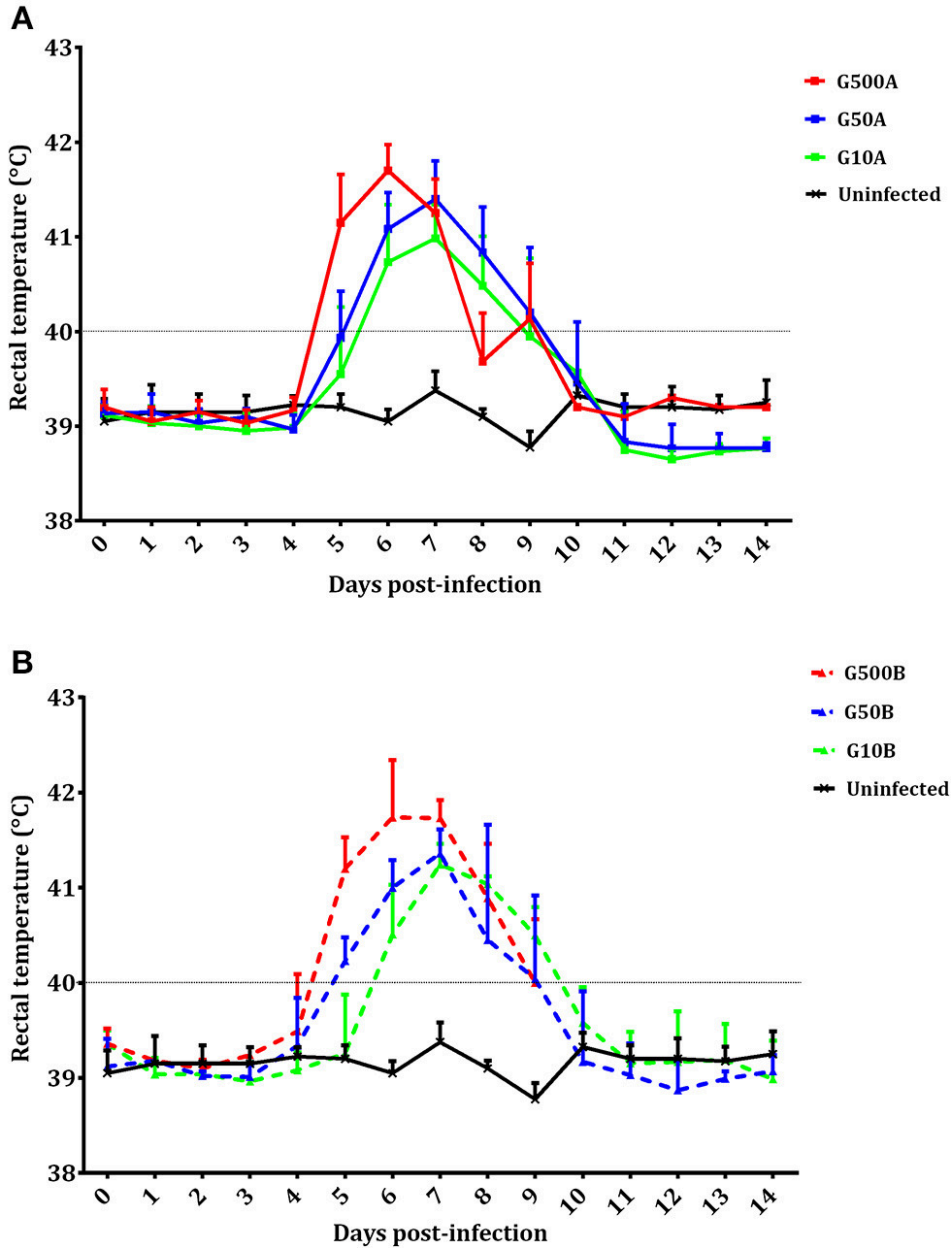
Rectal temperatures of ewes infected with TgShSp1 oocysts and the uninfected ones **(A)** and ewes infected with TgME49 oocysts and the uninfected ones **(B)** From day 7 pi onwards, some ewes aborted and were euthanized, and their data are not available. Each point represents the mean + S.D. at the different sampling times for each group.

No mortality was found in any sheep during the experiment. All ewes showed fever after infection. Significant increases in body temperature were found for 3–4 days after day 4 p.i. in all infected groups. From day 14 pi until the end of the experiment, no changes were detected in the infected groups. Likewise, compared to groups infected with 500 oocysts, 1 day of delay in the increase of rectal temperature (from day 5 to 6 pi) was found in groups infected with 50 and 10 oocysts (Figure 4). Differences in temperature increase between groups receiving the same dose of oocysts of the different *T. gondii* isolates were generally not found. As an exception, lower rectal temperatures were only found on day 8 pi in groups infected with 500 and 10 oocysts of TgShSp1 compared to groups receiving the same doses of TgME49 oocysts (G500A vs. G10A and G500B vs. G10B) (*P* < 0.05). The mean rectal temperature in the uninfected group remained below 39.5°C throughout the monitoring period.

Fetal/lamb mortality in groups receiving 500 oocysts was 100%. In groups receiving 50 and 10 oocysts, 68% of fetuses/lambs in G50A (in 6/6 ewes), and 66% in G10A (in 4/6 ewes) died after infection with TgShSp1, and 42% (in 2/5 ewes) of them died in both groups after infection with TgME49. Fetal/lamb mortality was increased with the oocyst doses. A lower fetal/lamb survival rate was found in the group infected with 500 TgME49 oocysts compared with that infected 50 oocysts (G500B vs. G50B) (*P* < 0.05). In addition, a significantly lower fetal/lamb mortality was found in those infected with 500 oocysts compared with those infected with 10 oocysts for both isolates (G500A and G500B compared to G10A and G10B, respectively) (*P* < 0.05). No differences in fetal/lamb mortality were found between groups infected with 50 and 10 oocysts (G50A and G50B compared to G10A and G10B, respectively). Comparing groups that received the same dose of sporulated oocysts but different isolates, no significant differences were found in the fetal/lamb survival rate. Concerning fetal death during pregnancy, all ewes challenged with 500 oocysts (G500A and G500B) aborted, and in the groups infected with lower doses, abortions were found in 3/6 and 2/5 pregnant ewes infected with 50 oocysts of TgShSp1 and TgME49, respectively, and in 1/6 and 1/5 pregnant ewes infected with 10 oocysts of TgShSp1 and TgME49, respectively (Table 2). Non-aborted dams gave birth between days 143 and 149 of pregnancy, except one ewe infected with 50 TgME49 oocysts, which gave birth prematurely on day 134. In G50A, 1 mummified fetus and 3 stillborn lambs were found, and in G50B, G10A, and G10B, one, eight and one stillborn lambs were delivered, respectively. Concerning morbidity in lambs born alive, weakness was found in 5 out of 5 and 1 out of 4 in groups infected with 50 oocysts, G50A and G50B, respectively, and in 2 out of 5 and 0 out of 4 in those infected with 10 oocysts, G10A and G10B, respectively. Therefore, morbidity in lambs born alive from the group infected with 50 TgShSp1 oocysts, G50A, was significantly higher than the corresponding TgME49 group, G50B (*P* < 0.05), whereas no significant differences in the number of weak lambs were found between lambs from groups infected with 10 oocysts or between lambs from groups receiving different doses of both isolates. Dams from the pregnancy control group gave birth two stillborn lambs and six healthy lambs between days 147 and 152 of pregnancy.

**Table 2:** Fetal/lamb mortality in sheep and percentages of placentomes/cotyledons or fetuses/lambs showing histological lesions and parasite detection.

**Group**	**Fetal/lamb mortality (%)**	**Clinical outcome**	**Number of ewes**	**Number of fetuses/lambs**	**Placentomes/cotyledons**		**Fetal brain**		**Fetal lung**	
					**H/E (%)**	**PCR (%)**	**H/E (%)**	**PCR (%)**	**H/E (%)**	**PCR (%)**
Group 500A (500 TgShSp1 oocysts)	100	Early abortions	5	11	NA	16	100	–	–	–
		Late abortions	1	1	100	100	100	100	–	100
Group 500B (500 TgME49 oocysts)	100	Early abortions	5	10	NA	–	100	–		–
Group 50A (50 TgShSp1 oocysts)	68	Early abortions	3	7	NA	16	100	–	–	14
		Stillborns/lambs	3	9	NA	100	63	60	–	100
Group 50B (50 TgME49 oocysts)	42	Early abortions	1	1	NA	–	100	–	–	–
		Late abortions	1	1	100	100	100	100	–	100
		Stillborns/lambs	3	5	NA	100	100	100	–	100
Group 10A (10 TgShSp1 oocysts)	66	Late abortions	1	2	100	100	100	100	–	100
		Stillborns/lambs	5	13	NA	100	69	100	–	100
Group 10B (10 TgME49 oocysts)	42	Early abortions	1	2	NA	–	100	–	–	–
		Stillborns/lambs	4	5	NA	75	60	60	–	50

*NA, not available*.

#### Parasite Detection and Burden in Placental and Fetal Tissues

##### Placental tissues

**Figure 5:**
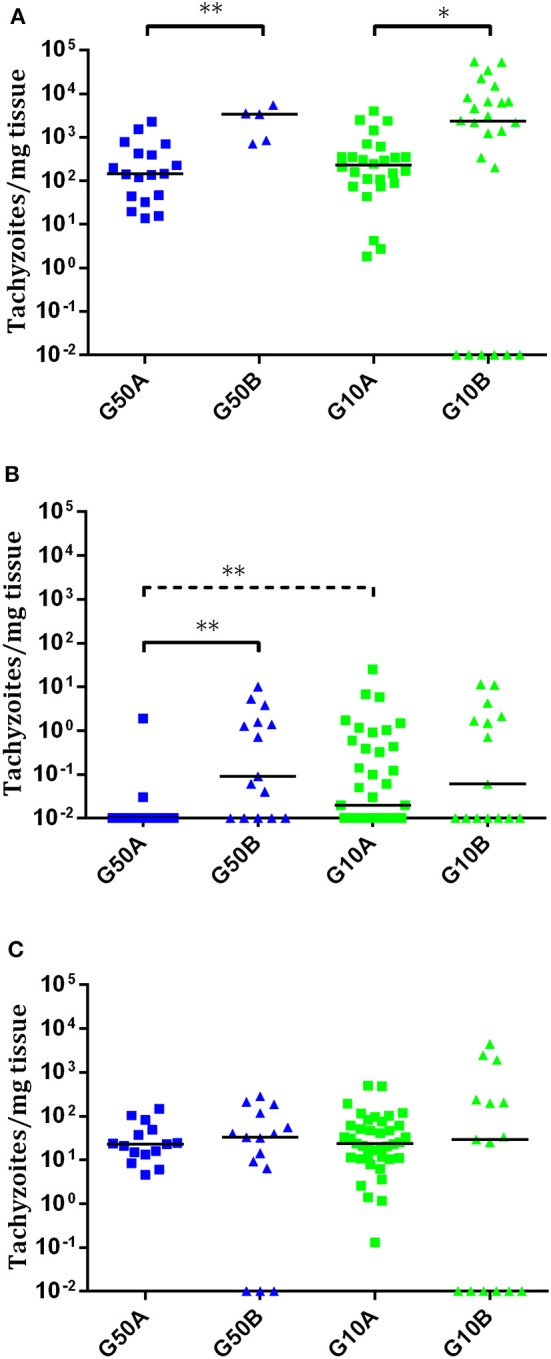
Dot-plot graphs of *T. gondii* burdens in cotyledons from ewes that gave birth **(A)** and brain **(B)** and lung **(C)** from stillborn lambs and live lambs from *T. gondii*-infected ewes. Each dot represents individual values of parasite burden (number of parasites per milligram of host tissue), and medians are represented as horizontal lines. Considering that the *T. gondii* detection limit by real-time PCR is 0.1 parasites, negative samples (0 parasites) were represented on the log scale as < 0.1 (i.e., 10^−2^). The unbroken line is used to indicate differences between isolates, and the dashed line (———) is used to indicate differences between doses. For significant differences between infected groups in each tissue, ^*^P < 0.05 and ^**^P < 0.01.

In ewes suffering early abortions, no *T. gondii* DNA was detected in placentomes in TgME49-infected animals (i.e., G500B, G50B, and G10B), and in ewes infected with TgShSp1, parasite DNA was only detected in one ewe infected with 500 oocysts (G500A), which aborted on day 9 pi (one positive placentome sample out of 30), and in one ewe infected with 50 oocysts (G50A), which aborted on day 10 pi (one positive placentome samples out of 18). In contrast, all placentomes from ewes showing late abortion were PCR-positive. In ewes that delivered stillbirths or live lambs, all cotyledons from TgShSp1-infected animals were PCR-positive. Among those challenged with TgME49 oocysts, 100% of cotyledon samples were positive in the group infected with 500 oocysts (G50B), while 75% of cotyledon samples were positive in the group infected with 10 oocysts G10B (Tables 2, S2). Concerning parasite burden (measured as the number of tachyzoites per milligram of tissue) in cotyledons from ewes that delivered stillbirths or live lambs, no differences were found between groups infected with 50 and 10 oocysts in any of the isolates. However, comparing both *T. gondii* isolates, parasite loads in cotyledons from ewes that gave birth in groups infected with 50 and 10 oocysts of TgShSp1 (groups G50A and G10A), both were lower compared to those infected with TgME49 (G50B and G10B) (*P* < 0.05) (Figure 5A).

##### Fetal tissues

In tissues from fetuses undergoing early abortion upon TgME49 infection, no *T. gondii* DNA was detected. The same was true for fetuses from early abortions after challenge with TgShSp1 oocysts, except for one positive fetal lung sample (1 positive sample out of 20) from one ewe that aborted on day 10 pi in one group infected with 50 oocysts (G50A) (Tables 2, S2).

In late abortions, *T. gondii* DNA was detected in every fetus and all organs analyzed (Table 2). The percentage of positive samples in every individual organ ranged from 33% in the brain from one fetus in G10A to 100% in the rest of the brains and lungs from late abortions (Table S2).

In all stillbirths and live lambs, *T. gondii* DNA was found in at least one of the studied organs, except in one stillborn lamb and one live lamb born from one ewe infected with 10 TgME49 oocysts (G10B) in which *T. gondii* DNA was not detected in any analyzed tissue (Table S2). Concerning parasite detection in the brain of stillbirths/live lambs born from ewes infected with TgME49 oocysts, more samples were PCR-positive in the group infected with 50 oocysts, G50B (91.6%; 11/12; 4 out of 4 fetuses) compared to the group infected with 10 oocysts, G10B (53.3%; 8/15; 3 out of 5 fetuses) (*P* < 0.05). In lambs from ewes infected with TgShSp1 oocysts, a lower number of brain samples were found to be PCR-positive in the group infected with 50 oocysts, G50A (33.3%; 5/15; 7 out of 9 fetuses), compared to the group infected with 10 oocysts, G10A (66.6%; 26/39; 13 out of 13 fetuses) (*P* < 0.05). Similarly, lower parasite burden in brain from lambs was found in the group infected with 50 TgShSp1 oocysts (G50A vs. G10A (*P* < 0.01). Comparing both isolates, parasite detection and parasite burden were higher in brain samples from lambs in the group infected with 50 oocysts of TgME49 compared to the corresponding TgShSp1 group G50B vs. G50A) (*P* < 0.01), while no significant differences were found between groups infected with 10 oocysts (G10A vs. G10B) (Figure 5B; Table 2; Table S2). In lung tissues from stillbirths/live lambs, all samples were PCR-positive in those groups infected with 50 oocysts (G50A and G50B). Additionally, 100% parasite detection was observed in the group infected with 10 oocysts of TgShSp1 (G10A), whereas a significantly lower parasite detection rate was found in the group infected with 10 oocysts of TgME49, G10B (60%; 9/15; 3 out of 6 animals) (*P* < 0.05). Comparison of the same oocyst dose from both isolates revealed no differences in parasite detection in lung samples from groups infected with 50 oocysts (G50B and G50A), but a higher parasite detection rate was found in the group infected with 10 TgShSp1 oocysts, G10A, compared to the group infected with 10 TgME49 oocysts, G10B (*P* < 0.001) (Table 2; Table S2). No differences in parasite burden in lungs from lambs were found between different doses or isolates (Figure 5C). Likewise, no differences in parasite detection or parasite burden were found in any fetal tissue between stillborn lambs and live lambs (data not shown). Samples from fetal tissues exhibiting DNA degradation and mummification were excluded from PCR analysis.

#### Histological Lesions and Lesion Quantification

The only evident histological lesions in the studied organs were found in the brain from fetuses/lambs and placenta. Only the placenta from late abortions detected through US was available for histological study. As in those cases of early abortions, lambing or delivery of stillbirths, it was too autolytic to allow proper histological evaluation.

In early abortions, multifocal areas of coagulative necrosis at the white matter (leukomalacia) were found in the brain from all the fetuses aborted in this period. In addition, no evident differences in the severity or number of lesions were noted between groups. In late abortions, lesions (multifocal necrotic placentitis) were found in all placentas studied. Likewise, there were brain lesions (multifocal non-purulent encephalitis) in all the fetuses from late abortions (Table 2).

In stillbirths/live lambs, glial foci with or without a central area of necrosis were observed in the brain. These lesions were found in lambs from all groups, with a prevalence between 60 and 100%, depending on the group (Table 2). When the percentages of brain lesions in lambs from ewes infected with TgME49 and TgShSp1 were compared, there were no differences between isolates or oocyst doses tested. Furthermore, there was no difference in the percentage of cases with brain lesions between stillbirths and live lambs (data not shown). Lesion quantification was carried out in brain samples from the stillbirths and live lambs, and no significant difference was found in the number of lesions, individual focus area or percentage of damaged area between groups (Figure S4).

#### Humoral Immune Responses

**Figure 6:**
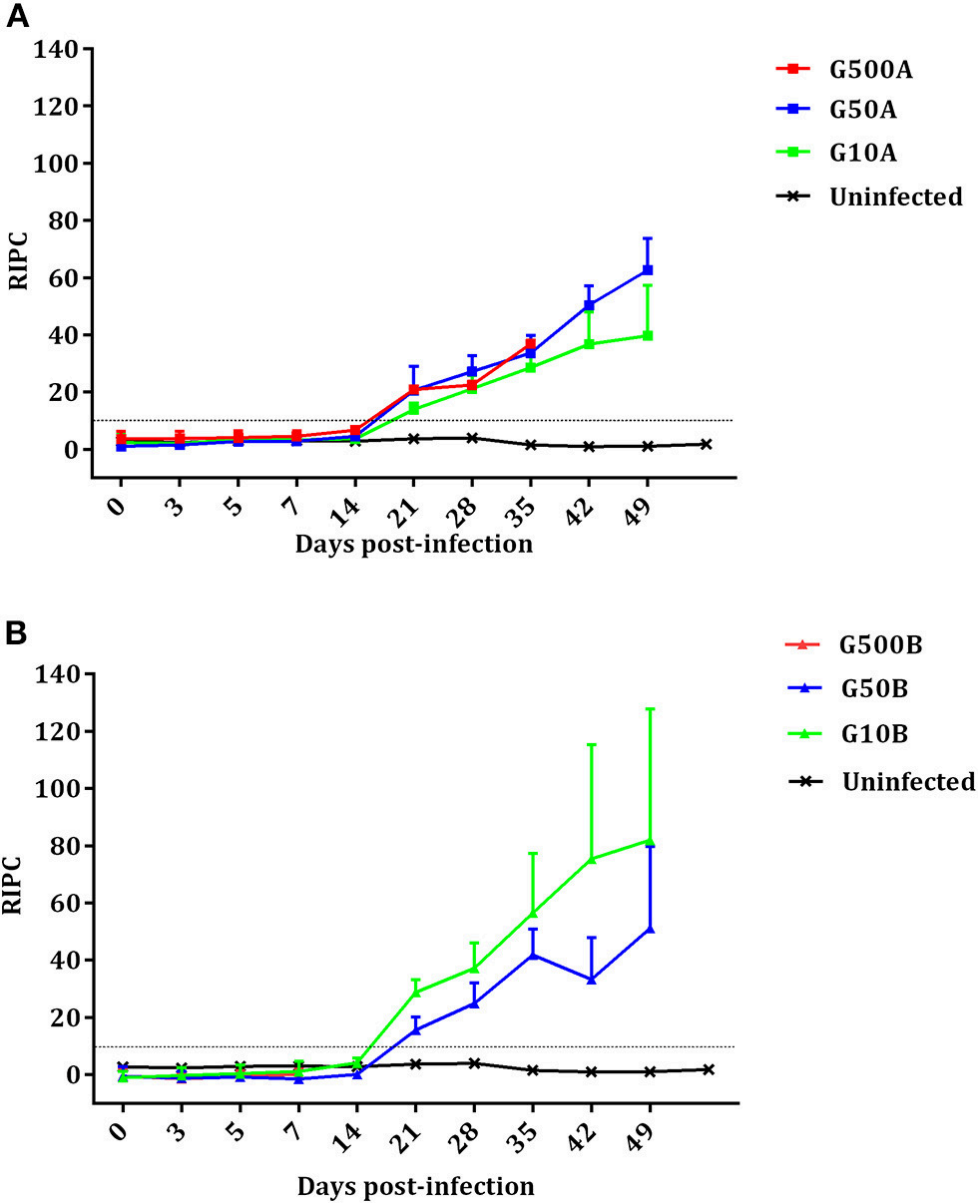
Kinetics of the antibody production in ewes infected with TgShSp1 oocysts and the uninfected ones **(A)** and ewes infected with TgShSp1 oocysts and the uninfected ones **(B)**. From day 7 pi onwards, some ewes aborted and were euthanized, and their data are not available. Each point represents the mean + S.D. at the different sampling times for each group. Serum levels of total IgG antibodies against *T. gondii* are expressed as a relative index percent (RIPC), according to the formula: RIPC = (OD405 sample – OD405 negative control)/(OD405 positive control – OD405 negative control) × 100.

The *Toxoplasma*-specific IgG antibody responses in dams are shown in Figure 6. No increase in IgG level compared to the uninfected group and no seroconversion was found in any of the ewes showing abortion during the acute phase of the infection. However, ewes with late abortion or those giving birth seroconverted on day 21 pi. In the group infected with 500 TgShSp1 oocysts, 500A, all ewes except one suffered early abortions, so this group was excluded from statistical analysis. From day 21 pi onwards, ewes infected with 50 and 10 oocysts seroconverted and exhibited higher IgG compared to the control group (*P* < 0.05). When analyzing the IgG levels of animals infected with TgShSp1 sporulated oocysts, no significant differences were found between ewes infected with 50 and 10 oocysts, G50A and G10A (Figure 6A). However, in animals infected with TgME49 sporulated oocysts, it is noteworthy that the group infected with 10 oocysts, G10B, had higher IgG on day 21 pi than the group infected with 50 oocysts, G50B (*P* < 0.01) (Figure 6B). Comparing groups receiving the same dose of sporulated oocysts, no significant differences in IgG level were found between groups infected with 50 oocysts, G50A and G50B. However, the group infected with 10 oocysts of TgShSp1, G10A, displayed lower IgG than that infected with TgME49, G10B, from day days 21 to 35 pi (*P* < 0.01). All uninfected control animals exhibited basal IgG levels within the reference range throughout the experimental study.

None of the fetuses that were aborted before day 11 pi had detectable IgG against *T. gondii* antigen. In contrast, fetuses undergoing late abortions were IgG-positive. Of the lambs born from TgShSp1-infected ewes, seven out of eight and nine out of twelve lambs were positive in groups infected with 50 and 10 TgShSp1 oocysts, G50A and G10A, respectively. Similarly, of the lambs born from TgME49-infected ewes, two out of three and 50% of lambs born from those groups infected with 50 and 10 TgME49 oocysts, G50B and G10B, respectively, were positive (Table S3). Specific IgG responses against parasite antigen were not detected in lambs from the uninfected group.

### Comparative Assessment of Congenital Infection in Mice and Sheep After Infection With TgShSp1 Oocysts

Offspring mortality after infection with 500 TgShSp1 oocysts occurred in sheep at a statistically higher rate compared to mice (*P* < 0.001), since in sheep all fetuses died and in mice only 50% of the pups died. Similarly, higher offspring mortality was found in sheep compared to mice after infections with intermediate (*P* < 0.0001) and low doses of oocysts (*P* < 0.01), since infection with 50 and 10 TgShSp1 oocysts triggered mortality in 68 and 66% of fetuses/lambs, whereas in mice mortality of 9 and 27% of the pups was observed after infection with 100 and 25 TgShSp1 oocysts, respectively.

Since no lambs were born in the group infected with 500 TgShSp1 oocysts, offspring morbidity in this group could not be assessed. However, in mice infected with 500 TgShSp1 oocysts, a decrease in pup body weight was found from day 14 pi onwards. Offspring from mice infected with 100 TgShSp1 oocysts showed a decrease in bodyweight from day 14 pi onwards, and all lambs born alive from ewes infected with 50 TgShSp1 exhibited weakness at birth. At low doses of oocysts, no body weight decrease was noted in pups born from mice infected with 25 TgShSp1 oocysts; however, 2 out 5 live lambs from ewes infected with 10 TgShSp1 oocysts were born with obvious weakness and impaired health.

All (100%) of the surviving mouse pups were PCR-positive in the brain, and 100% live lambs were seropositive or with a PCR-positive result in at least one tissue after infection with intermediate doses of oocysts (100 TgShSp1 oocysts in mice and 50 TgShSp1 oocysts in sheep). Additionally, 100% of the surviving pups were PCR-positive in the brain after infection with 500 TgShSp1 oocysts (high dose of oocysts). However, a statistically higher number of *T. gondii*-positive offspring were found in sheep compared to mice after infection with low doses of oocysts (*P* < 0.05), since 100% lambs were seropositive or with a PCR-positive result in at least one tissue after infection with 10 TgShSp1 oocysts and only 37% of the surviving pups were PCR-positive in the brain after infection with 25 TgShSp1 oocysts (Table 3).

**Table 3:** Summary of the outcome of the congenital infection in mice and sheep after infection with different doses of TgShSp1 oocysts.

**Doses of oocysts (Mice/Sheep)**	**Offspring mortality^a^ (Mice/Sheep)**	**Morbidity in the offspring^b^ (Mice/Sheep)**	**Vertical transmission^c^ (Mice/Sheep)**
High (500 oocysts/500 oocysts)	Medium/High^***^	High/NA	High/NA
Intermediate (100 oocysts/50 oocysts)	Low/High^****^	High/High	High/High
Low (25 oocysts/10 oocysts)	Low/Medium^**^	–/Medium	Medium/High^*^

a *Mortality of fetuses during pregnancy in sheep and of pups/lambs after birth in mice and sheep*.

b *Morbidity in the offspring was evaluated by decrease of the body weight of pups from day 14 pp in mice and by clinical signs in live lambs (weakness) at birth*.

c *In mice, PCR-positive brains in surviving pups at day 28 pp. In sheep, seropositive live lambs with a PCR-positive result in at least one tissue*.

*NA, not available. High, medium, low or minus (–) mean presence of clinical signs, offspring mortality, or vertical transmission of the parasite in >67, 66–34, < 33, and 0% of the fetuses/pups/lambs, respectively. Body weights lower than the uninfected group are considered high morbidity, while no difference compared to uninfected group is represented as minus (–)*.

* *P < 0.05*,

** *P < 0.01*,

*** P < 0.001, and

**** *P < 0.0001 significant differences between mice and sheep in the number of fetuses/pups/lambs died or in the number of surviving offspring infected with T. gondii*.

## Discussion

*Toxoplasma gondii* is an apicomplexan parasite that is distributed worldwide (Dubey, 2010). In Europe and North America, *T. gondii* isolates display a clonal population structure, with the vast majority of *T. gondii* isolates being grouped into three lineages, namely, types I, II and III (Howe and Sibley, 1995). Type II *T. gondii* is the most prevalent in all hosts in Europe, including sheep (Dumètre et al., 2006; Halos et al., 2010; Su et al., 2010). Previous studies in Europe have shown that *T. gondii* type II is associated with ovine abortion (Owen and Trees, 1999; Jungersen et al., 2002; Chessa et al., 2014). In Spain, type II is the most prevalent genotype in wild animals and cats (Montoya et al., 2008; Calero-Bernal et al., 2015), as well as in previously obtained ovine isolates (Fuentes, 1999). The TgShSp1 isolate belongs to genotype #3 (a type II variant, type II for nine alleles/type I for Apico), sharing genotype with the Prugniaud (PRU) isolate.

*Toxoplasma gondii* PRU isolates exhibit a similar genetic pattern to the *T. gondii* type II reference isolate, TgME49 (genotype #1, type II for the studied alleles) (Su et al., 2012). In addition, both type II isolates, TgME49, and PRU, activate the host cell transcription factor NF-κB, an integral component of the immune response to *T. gondii*, and they display identical GRA15 gene sequences, which is involved in NF-κB activation (Rosowski et al., 2011). TgME49 was isolated from sheep muscle in 1958 (Lunde and Jacobs, 1983) and has since then undergone long-term passaging in cell culture and mice (Sibley et al., 2002). Previous studies have demonstrated changes in biological characteristics of *T. gondii* isolates after passages in mice and cell culture (Frenkel et al., 1976; Lindsay et al., 1991; Harmer et al., 1996; Saraf et al., 2017). This fact has been widely studied in *T. gondii* type I isolates (Cesbron and Sabin, 1994; Villard et al., 1997; Dubey et al., 1999; Mavin et al., 2004; Khan et al., 2009). However, whether these changes also occur in type II isolates, and how they compare to recently obtained isolates, remains unknown. Increased growth *in vitro* can be found after repeated passages (Yano et al., 1987). The dramatic differences observed during *in vitro* growth of TgME49 and TgShSp1 might reflect the highly different passage history of the two isolates. Plaque formation is commonly used to measure growth of *T. gondii*, and this process is the result of several events, including invasion, growth, egress, and migration (Roos et al., 1994). Notably, TgME49 tachyzoites formed plaques at 4 days pi, but TgShSp1 did not. The finding that TgShSp1 did not form plaques *in vitro* could have resulted from the limited growth rate, associated with a higher capacity of bradyzoite conversion, as previously described (Khan et al., 2009). This was confirmed by monitoring spontaneous cyst formation through labeling with the fluorescent lectin DBL, demonstrating tissue cyst formation under standard cell culture procedures in TgShSp1 that was greater than TgME49. Due to its high passage number in cell culture or mice, our TgME49 isolate may not accurately represent natural virulence traits of the type II lineage, which suggests that comparisons of phenotypes between *T. gondii* isolates should be conducted using low-passage stocks.

Traditionally, mouse models are utilized to evaluate virulence by monitoring survival after experimental infection. Type I isolates are highly virulent in mice (LD_100_ of 1 tachyzoite), whereas types II and III exhibit median lethal doses (LD_50_) that range from 10^2^ to 10^5^ (Saeij et al., 2006). Conventionally, TgME49 is a cystogenic type II isolate with low virulence in mice (Ferreira et al., 2001; Gavrilescu and Denkers, 2001; Oliveira et al., 2016). Intraperitoneal inoculation of 10^3^ and 5 × 10^4^ TgME49 tachyzoites intraperitoneally has not caused mortality (Ferreira et al., 2001; Oliveira et al., 2016). However, in this study, TgME49 displayed a LD_50_ of 10^3^, so the virulence of our TgME49 could be considered stronger than previous descriptions (Ferreira et al., 2001; Oliveira et al., 2016). Enhanced virulence in mice for *T. gondii* strains maintained for several passages has also been reported previously (Shimizu et al., 1967; Sibley and Boothroyd, 1992; Frenkel and Ambroise-Thomas, 1996). Hence, it seems logical to speculate that results from studies using laboratory isolates should be validated with more recent isolates before they can be extrapolated as general features of the respective lineage. In contrast to TgME49, mice inoculated with tachyzoites of the recently obtained type II isolate TgShSp1 exhibited only moderate, low-level clinical signs, but no mortality (LD_50_ > 10^5^), similar to what was described earlier after intraperitoneal inoculation of Swiss Webster mice with 10^3^ tachyzoites of a PRU isolate (Wang et al., 2013). In addition, although oocysts are considered more virulent than tachyzoites in mice (Dubey and Frenkel, 1973), no mortality in adult mice was found after infection with oocysts of TgShSp1 in pregnant and non-pregnant mice, suggesting very low virulence in mice.

In the present work, we also investigated congenital toxoplasmosis in pregnant mice by inoculating them orally with different doses of TgShSp1 oocysts. The risk of congenital toxoplasmosis depends on the virulence of the parasite (Tenter et al., 2000). Based on the previously established toxoplasmosis model using TgME49 oocysts (Müller et al., 2017), we infected mice at day 7 post-mating, which represents the beginning of the second term of gestation. Few mice infected with TgShSp1 oocysts showed mild clinical signs, contrary to the large number of mice succumbing to infection after the same oocyst doses of TgME49 (Müller et al., 2017). There is a possibility that there has been a selection toward increased virulence within TgME49 due to the successive passages, as explained above but also due to the sulfadimidine treatment that was applied in mice used for infection of cats to generate TgME49 oocysts used in this study (Müller et al., 2017). This sulfonamide treatment could act as a bottleneck in selecting tachyzoites with a faster replication and therefore increasing the virulence in mice of the final TgME49 parasites. Unlike what was observed for TgME49 (Müller et al., 2017), with which a clear effect on pregnancy rate was found after infection with 2,000 oocysts and on litter size after infection with 500 oocysts, infection with TgShSp1 oocysts did not result in alteration of pregnancy rate or litter size. Likewise, while 92% of the pups died after infection of dams with 25 TgME49 oocysts (Müller et al., 2017), a significant decrease in pup survival was found only after infection with 2,000 and 500 TgShSp1 oocysts, where there was mortality in 50% of the pups. In conclusion, infection of mice with TgShSp1 oocysts at mid-pregnancy did not generate severe clinical signs in adult mice, but infection doses of 2,000 and 500 oocysts in the dams resulted in mortality of 50% of the pups and decreased body weight in surviving pups, and 100% vertical transmission occurred with doses of up to 100 oocysts.

Sheep are a relevant host of the parasite and could suffer abortions when primo infected during gestation (Vargas-Villavicencio et al., 2016). Fetal/lamb mortality was similar for both isolates. However, those ewes infected with 10 and 50 TgME49 oocysts and that delivered stillbirths/live lambs exhibited higher parasite load in cotyledons than those infected with the same doses of TgShSp1 oocysts. Similarly, a higher parasite load was found in the brain from lambs born in the group infected with 50 TgME49 oocysts compared to the corresponding TgShSp1 group. Therefore, it is tempting to hypothesize that the enhanced virulence of our TgME49 contributed to the abovementioned effects. Comparing different doses of infection, there is a correlation between the dose of infection and the rate of early abortions, as previously suggested (Mévélec et al., 2010; Benavides et al., 2017). Infection with 500 oocysts triggered abortion in all fetuses, similar to previous experimental infections in pregnant sheep at mid-pregnancy using 2,000 oocysts of M1 and M4 isolates (Owen et al., 1998; Castaño et al., 2014). After infection with 50 TgShSp1 oocysts or 50 TgME49 oocysts, 68 and 42% of fetuses/lambs died, respectively, similar to what was previously reported after infection with 50 M4 oocysts (Castaño et al., 2014). The occurrence of abortions after infection with 10 oocysts was low, but large numbers of stillbirths and weak lambs were found, mainly in the 10 TgShSp1 oocysts group. There seems to be a correlation between the presence of the parasite and the occurrence of stillbirths, since stillbirths from the group infected with 10 TgShSp1 oocysts exhibited higher parasite detection and load in the brain than those in the group infected with 50 TgShSp1 oocysts. Regardless of the isolate or dose, no differences were found in the congenital infection of lambs born, since vertical transmission was found in all them except in one stillborn lamb and one live lamb born from one ewe infected with 10 TgME49 oocysts. Likewise, no differences were found between doses or isolates with respect to brain lesion presence and lesion severity in lambs born.

Most of the experimental studies in pregnant sheep carried out so far used M1, M3, and M4 *T. gondii* type II isolates for infection (Dubey, 2009; Castaño et al., 2014). However, although numerous experimental infections were also carried out in mice with these isolates (Nicoll et al., 1997; Owen et al., 1998; Hamilton et al., 2018), their virulence in mice models has not been studied in depth. Therefore, the correlation between virulence in mice and outcome of experimental infections in pregnant sheep has not been elucidated. Despite the clear differences in body weight between mice and sheep, in the current study, similar doses of oocysts were used to compare both hosts. None of the adult mice challenged with 25 TgShSp1 oocysts exhibited clinical signs, whereas all ewes challenged with 10 TgShSp1 oocysts had fever. Therefore, morbidity in sheep seems to be higher than in mice. In addition, no mortality was observed in adult mice or sheep infected with TgShSp1 oocysts. When comparing the congenital infection after challenge at mid-pregnancy between both hosts, 50% mortality was caused in mice by infection with 500 TgShSp1 oocysts, whereas in sheep infection with the same oocyst dose caused mortality in all fetuses. In brief, mice seem to be less susceptible to offspring mortality than sheep, despite the fact that vertical transmission was similar in both species. High vertical transmission and low offspring mortality could be an evolutionary strategy of the parasite to generate a large infected offspring group in mice, one of the most relevant hosts of *T. gondii* (Müller and Howard, 2016).

There are several differences between mice and sheep that could underlie the differences found in this study. The histological structure of the placenta is very different between mice and sheep (Entrican, 2002), and although maternal blood and fetal tissue are closer in mice, allowing an easy crossing of tachyzoites but also of antibodies, the longer period of gestation, the lack of maternal antibodies crossing the placental barrier and fewer fetuses may facilitate vertical transmission in sheep. In addition, host genetics are likely important in determining susceptibility and severity of infection (Howe et al., 1996; Müller and Howard, 2016). Small rodents, natural intermediate hosts, are often exposed to a higher dose and more virulent parasites than other groups of mammals. It may therefore be that Toll-Like-Receptors (TLR)11 and TLR12 and the polymorphism of immunity-related GTPases (IRG proteins) have been positively selected in rodents, because of their critical importance in host resistance against high infection loads or more virulent clones of *T. gondii*. In mice, TLR11 and TLR12 on dendritic cells detect the apicomplexan actin-binding protein profilin leading to the secretion of interleukin 12 (IL12), which can subsequently induce production of IFNγ by T cells. IFNγ induces a variety of parasiticidal mechanisms, which in mice are dominated by upregulation of the IRGs (Gazzinelli et al., 2014). IRGs can destroy the vacuole in these parasites live and subsequently the parasite itself. Considering the ubiquity of *T. gondii* in nature, it is intriguing that genes encoding TLR11, TLR12, and IRG proteins are not found in many mammalian species (Gazzinelli et al., 2014). Although further studies are needed, and despite the influence of genetic polymorphisms in ovine abortions (Darlay et al., 2011), this fact could render sheep less resistant. Similarly, differences in immune cell populations may influence the pathogenesis of toxoplasmosis in these hosts. γδ T cells, which rapidly recognize and respond to non-processed antigens and seem to have an important role in *T. gondii* infection (Egan et al., 2005), represent a relevant subset of circulating T cells in sheep compared to mice (Holderness et al., 2013). Further studies are needed to characterize the cellular and molecular bases contributing to transmission dynamics and disease in different hosts of *T. gondii* (Dubremetz and Lebrun, 2012; Hunter and Sibley, 2012).

In conclusion, we have demonstrated that infection with tachyzoites and oocysts of the type II *T. gondii* isolate TgShSp1 in mice does not cause mortality, but this isolate is efficiently vertically transmitted in pregnant mice, and compared to sheep, it triggers lower offspring mortality and morbidity. Thus, at least for this isolate, the disease caused in pregnant mice and offspring is not a reliable predictor/indicator for disease caused in pregnant sheep at mid-gestation. Whether this conclusion is also valid for other type II *T. gondii* strains needs to be addressed in future studies. In addition, our results suggest that the laboratory isolate TgME49 exhibits an enhanced virulence due to successive passages in cell culture and mice. Thus, virulence traits may have been modified, and it might be advisable to use low-passage isolates in experimental studies, as these probably provide a more realistic picture of the true nature of the parasite biology in the field.

## Author Contributions

IF, JR-C, VP, AH, LO-M, and JB conceived the study and participated in its design. LO-M coordinated the isolation, *in vitro* and mouse studies, and JB and LO-M coordinated the studies in sheep. RS-S, LO-M, and JB wrote the manuscript, with result interpretation and discussion inputs from IF, JR-C, and AH. JR-C, JM-G, and JB carried out the isolation of TgShSp1. RS-S and JR-C carried out *in vitro* experiments. LF selected sheep and executed the reproductive program. RS-S, IF, JR-C, and JM prepared tachyzoites or oocysts and performed the infections. RS-S, DG-E, NA-V, JM-G, AA-M, VP, and JB participated in inoculation and clinical examination of animals, performed necropsies and sampling of the animals and performed histopathological analyses. RS-S performed PCR and qPCR analyses, serological assays, and statistical analysis and interpreted the results. All authors read and approved the final manuscript.

### Conflict of Interest Statement

The authors declare that the research was conducted in the absence of any commercial or financial relationships that could be construed as a potential conflict of interest.

## Supplementary Material

Figure S1 Box-plots showing IgG serum titers generated in dams **(A)** and non-pregnant mice **(B)** infected with TgShSp1 oocysts and the uninfected group. Graphs represent the median percentage, the lower and upper quartiles (boxes) and minimum and maximum values (whiskers). For significant differences in serum titres, ^*^*P* < 0.05 and ^**^*P* < 0.01.

Figure S2 Dot-plot graphs of *T. gondii* burdens in brain from dams **(A)** and non-pregnant mice **(B)**. Each dot represents individual values of parasite burden (number of parasites per microgram of DNA), and medians are represented as horizontal lines.

Figure S3 Dot-plot graphs of *T. gondii* burdens in lung from dams **(A)** and non-pregnant mice **(B)**. Each dot represents individual values of parasite burden (number of parasites per microgram of DNA), and medians are represented as horizontal lines.

Figure S4 Dot-plots showing number of lesions **(A)**, individual focus area **(B)**, and percentage of damaged area **(C)** in the brains from stillborn lambs and live lambs. Each dot represents individual, and medians are represented as horizontal lines.

Table S1 Clinical signs, serological titers, and parasite detection in mice intraperitoneally infected with TgME49 and TgShSp1 tachyzoites.

Table S2 Individual frequency of parasite DNA detection in infected animals.

Table S3 Individual serological titers in fetuses/lambs from infected ewes.
