# Supplementary material for: Virulence in Mice of a Toxoplasma gondii Type II Isolate Does Not Correlate With the Outcome of Experimental Infection in Pregnant Sheep
Source: Front Cell Infect Microbiol. 2019 Jan 4;8:436. doi: 10.3389/fcimb.2018.00436 (PMC6328472; doi:10.3389/fcimb.2018.00436)
Supplement: Table S1 — Clinical signs, serological titers, and parasite detection in mice intraperitoneally infected with TgME49 and TgShSp1 tachyzoites. [file Table_1.DOCX]

**Table S1. Clinical signs, serological titres and parasite detection in mice intraperitoneally infected with TgME49 and TgShSp1 tachyzoites.**

| **Dose of tachyzoites** | **Mice ref.** | **Clinical signs (dpi)*** | **IFAT titre** | **PCR** | |
| --- | --- | --- | --- | --- | --- |
|  |  |  |  | **Brain** | **Lung** |
| **10^5^ TgShSp1** | **1** | 1 (4) | 1:400 | + | - |
|  | **2** | 1 (5) | 1:800 | + | + |
|  | **3** | 1 (7) | 1:1600 | + | + |
|  | **4** | 1 (9) | 1:800 | + | + |
|  | **5** | 1 (10) | 1:1600 | + | + |
| **10^5^ TgME49** | **1** | 3 (11)^T^ | Negative | + | + |
|  | **2** | 3 (8)^T^ | NA | + | + |
|  | **3** | 2 (8) | 1:800 | + | + |
|  | **4** | 2 (8) | 1:800 | + | + |
|  | **5** | 2 (8) | 1:400 | + | + |
| **10^4^ TgShSp1** | **1** | 2 (10) | 1:800 | + | + |
|  | **2** | 1 (8) | 1:800 | + | + |
|  | **3** | 1 (8) | 1:1600 | + | + |
|  | **4** | 1 (9) | 1:800 | + | + |
|  | **5** | 0 | 1:400 | + | - |
| **10^4^ TgME49** | **1** | 3 (10)^T^ | Negative | + | + |
|  | **2** | 2 (9) | 1:200 | + | - |
|  | **3** | 3 (14)^T^ | Negative | + | + |
|  | **4** | 2 (9) | 1:200 | + | + |
|  | **5** | 2 (9) | 1:800 | + | - |
| **10^3^ TgShSp1** | **1** | 1 (6) | 1:800 | + | - |
|  | **2** | 1 (6) | 1:400 | + | + |
|  | **3** | 1 (7) | 1:400 | + | + |
|  | **4** | 1 (9) | 1:1600 | + | + |
|  | **5** | 1 (9) | 1:800 | + | - |
| **10^3^ TgME49** | **1** | 4 (33)^T^ | 1:25 | + | + |
|  | **2** | 4 (13)^T^ | Negative | + | + |
|  | **3** | 2 (10) | 1:400 | + | + |
|  | **4** | 2 (10) | 1:400 | + | - |
|  | **5** | 3 (14)^T^ | NA | + | + |
| **10^2^ TgShSp1** | **1** | 1 (7) | 1:800 | + | - |
|  | **2** | 1 (8) | 1:1600 | + | + |
|  | **3** | 1 (8) | 1:1600 | + | + |
|  | **4** | 1 (9) | 1:3200 | + | + |
|  | **5** | 1 (13) | 1:800 | + | + |
| **10^2^ TgME49** | **1** | 2 (14) | 1:200 | + | + |
|  | **2** | 4 (13)^T^ | Negative | + | + |
|  | **3** | 2 (14) | 1:100 | + | + |
|  | **4** | 2 (14) | 1:100 | + | + |
|  | **5** | 2 (14) | 1:200 | + | - |

| **Dose of tachyzoites** | **Mice ref.** | **Clinical signs (dpi)*** | **IFAT titre** | **PCR** | |
| --- | --- | --- | --- | --- | --- |
|  |  |  |  | **Brain** | **Lung** |
| **10 TgShSp1** | **1** | 1 (7) | 1:800 | + | + |
|  | **2** | 0 | 1:800 | + | + |
|  | **3** | 0 | 1:800 | + | + |
|  | **4** | 0 | 1:800 | + | + |
|  | **5** | 0 | 1:1600 | + | + |
| **10 TgME49** | **1** | 2 (14) | 1:100 | + | - |
|  | **2** | 2 (14) | 1:100 | + | - |
|  | **3** | 4 (24)^T^ | Negative | + | + |
|  | **4** | 2 (14) | 1:100 | + | - |
|  | **5** | 2 (14) | 1:50 | + | - |
| **1 TgShSp1** | **1** | 0 | Negative | - | - |
|  | **2** | 0 | Negative | - | - |
|  | **3** | 0 | Negative | - | - |
|  | **4** | 0 | Negative | - | - |
|  | **5** | 0 | Negative | - | - |
| **1 TgME49** | **1** | 0 | Negative | - | - |
|  | **2** | 0 | Negative | - | - |
|  | **3** | 0 | Negative | - | - |
|  | **4** | 0 | Negative | - | - |
|  | **5** | 0 | Negative | - | - |

^*^ Clinical signs compatible with toxoplasmosis. Scores of 0 (no alterations), 1 (ruffled coat), 2 (rounded back), 3 (noticeable loss of body condition/severe weight loss) or 4 (nervous signs such as activity decrease, hind limb paralysis, walking in circles or head tilt). In brackets, day post infection when mice exhibited clinical signs.

^T^ Mice showing clinical scores of 3 or 4 were euthanized to limit unnecessary suffering.

NA: not available
