# Supplementary material for: Virulence in Mice of a Toxoplasma gondii Type II Isolate Does Not Correlate With the Outcome of Experimental Infection in Pregnant Sheep
Source: Front Cell Infect Microbiol. 2019 Jan 4;8:436. doi: 10.3389/fcimb.2018.00436 (PMC6328472; doi:10.3389/fcimb.2018.00436)
Supplement: Table S2 — Individual frequency of parasite DNA detection in infected animals. [file Table_2.DOCX]

**Table S2. Individual frequency of parasite DNA detection** **in infected animals.**

| **Group** | **Ewe ref.** | | **Foetal death (dpi)^a^** | **Placentomes/cotyledons^b^** | **Foetal/ lamb ref** | **Foetal tissues** | | | | | | | | |
| --- | --- | --- | --- | --- | --- | --- | --- | --- | --- | --- | --- | --- | --- | --- |
|  |  |  |  |  |  | **Brain** | | | | | **Lung** |  |  |  |
| **G500A**  **(500 TgShSp1 oocysts)** | **500A.1** | | 9 | + | 500A.1F1 | | | - | | - | | | |  |
|  | **500A.2** | | 36 | +++ | 500A.2F1 | | | +++ | | +++ | | | |  |
|  | **500A.3** | | 8 | - | 500A.3F1 | | | - | | - | | | |  |
|  | **500A.4** | | 9 | - | 500A.4F1 | | | - | | - | | | |  |
|  |  | |  |  | 500A.4F2 | | | - | | - | | | |  |
|  |  | |  |  | 500A.4F3 | | | - | | - | | | |  |
|  | **500A.5** | | 8 | - | 500A.5F1 | | | - | | - | | | |  |
|  | **500A.6** | | 8 | - | 500A.6F1 | | | - | | - | | | |  |
|  |  | |  |  | 500A.6F2 | | | - | | - | | | |  |
|  |  | |  |  | 500A.6F3 | | | - | | - | | | |  |
|  |  | |  |  | 500A.6F4 | | | - | | - | | | |  |
|  |  | |  |  | 500A.6F5 | | | - | | - | | | |  |
| **G500B (500 TgME49 oocysts)** | **500B.1** | | 8 | - | 500B.1F1 | | | - | | - | | | |  |
|  |  | |  |  | 500B.1F2 | | | - | | - | | | |  |
|  | **500B.2** | | 9 | - | 500B.2F1 | | | - | | - | | | |  |
|  |  | |  |  | 500B.2F2 | | | - | | - | | | |  |
|  |  | |  |  | 500B.2F3 | | | - | | - | | | |  |
|  | **500B.3** | | 8 | - | 500B.3F1 | | | - | | - | | | |  |
|  |  | |  |  | 500B.3F2 | | | - | | - | | | |  |
|  | **500B.4** | | 9 | - | 500B.4F1 | | | - | | - | | | |  |
|  | **500B.5** | | 9 | - | 500B.5F1 | | | - | | - | | | |  |
|  |  | |  |  | 500B.5F2 | | | - | | - | | | |  |
| **G50A**  **(50 TgShSp1 oocysts)** | | **50A.1** | a | +++ | 50A.1F1 | | | - | | +++ | | | |  |
|  |  |  |  |  | 50A.1F2 | | γ | | γ | | | |  |  |
|  |  |  |  |  | 50A.1F3 | | β | | β | | | |  |  |
|  |  | **50A.2** | 10 | + | 50A.2F1 | | - | | + | | | |  |  |
|  |  |  |  |  | 50A.2F2 | | - | | - | | | |  |  |
|  |  |  |  |  | 50A.2F3 | | - | | - | | | |  |  |
|  |  | **50A.3** | 9 | - | 50A.3F1 | | | - | | - | | | |  |
|  |  |  |  |  | 50A.3F2 | | | - | | - | | | |  |
|  |  | **50A.4** | a | +++ | 50A.4F1 | | | ++ | | +++ | | | |  |
|  |  |  |  |  | 50A.4F2 | | | ++ | | +++ | | | |  |
|  |  | **50A.5** | a |  | 50A.4F3 | | | γ | | γ | | | |  |
|  |  |  |  | +++ | 50A.5F1 | | | - | | +++ | | | |  |
|  |  |  |  |  | 50A.5F2 | | | + | | +++ | | | |  |
|  | | **50A.6** | 10 | - | 50A.6F1 | | | - | | - | | | |  |
|  | |  |  |  | 50A.6F2 | | | - | | - | | | |  |
| **G50B**  **(50 TgME49 oocysts)** | | **50B.1** | 35 | +++ | 50B.1F1 | | | +++ | | +++ | | | |  |
|  |  | **50B.2** | a | NA | 50B.2F1 | | | +++ | | +++ | | | |  |
|  |  | **50B.3** | 11 | - | 50B.3F1 | | | - | | - | | | |  |
|  |  | **50B.4** | a | +++ | 50B.4F1 | | | +++ | | +++ | | | |  |
|  |  |  |  |  | 50B.4F2 | | | +++ | | +++ | | | |  |
|  |  | **50B.5** | a | +++ | 50B.5F1 | | | ++ | | +++ | | | |  |

| **Group** | **Ewe ref.** | **Foetal death (dpi)^a^** | **Placentomes/cotyledons^b^** | **Foetal/ lamb ref** | | **Foetal tissues** | | | |  |
| --- | --- | --- | --- | --- | --- | --- | --- | --- | --- | --- |
|  |  |  |  |  |  | **Brain** | | | **Lung** |  |
| **G10A (10 TgShSp1 oocysts)** | **10A.1** | 49 | +++ | | 10A.1F1 | | γ | γ | | |
|  |  |  |  | | 10A.1F2 | | + | +++ | | |
|  | **10A.2** | a | +++ | | 10A.2F1 | | ++ | +++ | | |
|  |  |  |  | | 10A.2F2 | | + | +++ | | |
|  | **10A.3** | a | +++ | | 10A.3F1 | | +++ | +++ | | |
|  |  |  |  | | 10A.3F2 | | ++ | +++ | | |
|  |  |  |  | | 10A.3F3 | | ++ | +++ | | |
|  | **10A.4** | a | +++ | | 10A.4F1 | | +++ | +++ | | |
|  |  |  |  | | 10A.4F2 | | ++ | +++ | | |
|  | **10A.5** | a | +++ | | 10A.5F1 | | + | +++ | | |
|  |  |  |  | | 10A.5F2 | | + | +++ | | |
|  | **10A.6** | a | +++ | | 10A.6F1 | | +++ | +++ | | |
|  |  |  |  | | 10A.6F2 | | ++ | +++ | | |
|  |  |  |  | | 10A.6F3 | | + | +++ | | |
|  |  |  |  | | 10A.6F4 | | +++ | +++ | | |
| **G10B (10 TgME49 oocysts)** | **10B.1** | 11 | - | | 10B.1F1 | | - | - | | |
|  |  |  |  | | 10B.1F2 | | - | - | | |
|  | **10B.2** | a | +++ | | 10B.2F1 | | ++ | +++ | | |
|  | **10B.3** | a | - | | 10B.3F1 | | - | - | | |
|  |  |  |  | | 10B.3F2 | | - | - | | |
|  | **10B.4** | a | +++ | | 10B.4F1 | | +++ | +++ | | |
|  | **10B.5** | a | +++ | | 10B.5F1 | | +++ | +++ | | |

^a^ Day post-challenge when foetal death was detected by ultrasonography. The remaining ewes (*a*) delivered stillbirths/live lambs.

^b^ Placentomes in ewes that aborted and cotyledons in ewes that gave birth;

γ Samples from foetal tissues exhibiting DNA degradation were excluded.

β Mummified foetuses were not evaluated.

dpi: days post-infection

NA: not available

Plus (+++, ++, +) and minus (-) signs represent PCR detection in >67%, 66-34%, <33% and 0% of samples analysed, respectively.
