# Supplementary material for: Virulence in Mice of a Toxoplasma gondii Type II Isolate Does Not Correlate With the Outcome of Experimental Infection in Pregnant Sheep
Source: Front Cell Infect Microbiol. 2019 Jan 4;8:436. doi: 10.3389/fcimb.2018.00436 (PMC6328472; doi:10.3389/fcimb.2018.00436)
Supplement: Table S3 — Individual serological titers in fetuses/lambs from infected ewes. [file Table_3.DOCX]

**Table S3. Individual serological titres in foetuses/lambs from infected ewes.**

| **Group** | **Ewe ref.** | | | **Foetal death (dpi)^a^** | | **Foetus/Lambs ref.** | | | **FL or Sera titre** | |
| --- | --- | --- | --- | --- | --- | --- | --- | --- | --- | --- |
| **G500A**  **(500 TgShSp1 oocysts)** | **500A.1** | | | 9 | | 500A.1F1 | | | - | |
|  | **500A.2** | | | 36 | | 500A.2F1 | | | 1:64 | |
|  | **500A.3** | | | 8 | | 500A.3F1 | | | - | |
|  | **500A.4** | | | 9 | | 500A.4F1 | | | - | |
|  |  |  |  |  |  | 500A.4F2 | | | - | |
|  |  |  |  |  |  | 500A.4F3 | | | - | |
|  | **500A.5** | | | 8 | | 500A.5F1 | | | - | |
|  | **500A.6** | | | 8 | | 500A.6F1 | | | - | |
|  |  |  |  |  |  | 500A.6F2 | | | - | |
|  |  |  |  |  |  | 500A.6F3 | | | - | |
|  |  | | |  | | 500A.6F4 | | | - | |
|  |  | | |  | | 500A.6F5 | | | - | |
| **G500B (500 TgME49 oocysts)** | **500B.1** | | | 8 | | 500B.1F1 | | | - | |
|  |  | | |  | | 500B.1F2 | | | - | |
|  | **500B.2** | | | 9 | | 500B.2F1 | | | - | |
|  |  | | |  | | 500B.2F2 | | | - | |
|  |  | | |  | | 500B.2F3 | | | - | |
|  | **500B.3** | | | 8 | | 500B.3F1 | | | - | |
|  |  | | |  | | 500B.3F2 | | | - | |
|  | **500B.4** | | | 9 | | 500B.4F1 | | | - | |
|  | **500B.5** | | | 9 | | 500B.5F1 | | | - | |
|  |  | | |  | | 500B.5F2 | | | - | |
|  | | | **50A.1** | a | | 50A.1F1 | | | - | |
|  | |  | | |  | | 50A.1F2 | 1:8^ | |  |
|  | |  | | |  | | 50A.1F3 | NA | |  |
|  | | **50A.2** | | | 10 | | 50A.2F1 | - | |  |
|  | |  | | |  | | 50A.2F2 | - | |  |
|  | |  | | |  | | 50A.2F3 | - | |  |
|  | | **50A.3** | | | 9 | | 50A.3F1 | - | |  |
| **G50A (50 TgShSp1 oocysts)** | |  | | |  | | 50A.3F2 | - | |  |
|  | | **50A.4** | | | a | | 50A.4F1 | 1:800 | |  |
|  | |  | | |  | | 50A.4F2 | 1:100 | |  |
|  | |  | | |  | | 50A.4F3 | 1:16^ | |  |
|  | | **50A.5** | | | a | | 50A.5F1 | 1:100 | |  |
|  | |  | | |  | | 50A.5F2 | 1:100 | |  |
|  | |  | | |  | | 50A.5F3 | 1:16^ | |  |
|  | | **50A.6** | | | 10 | | 50A.6F1 | - | |  |
|  | |  | | |  | | 50A.6F2 | - | |  |
| **G50B (50 TgME49 oocysts)** | | **50B.1** | | | 35 | | 50B.1F1 | 1:128 | |  |
|  |  | **50B.2** | | | a | | 50B.2F1 | -^ | |  |
|  |  | **50B.3** | | | 11 | | 50B.3F1 | - | |  |
|  |  | **50B.4** | | | a | | 50B.4F1 | 1:400 | |  |
|  |  |  | | |  | | 50B.4F2 | 1:200 | |  |
|  |  | **50B.5** | | | a | | 50B.5F1 | NA* | |  |

| **Group** | **Ewe ref.** | **Foetal death (dpi)** | **Foetus/Lambs ref.** | **FL or Sera titre** |
| --- | --- | --- | --- | --- |
|  | **10A.1** | 49 | 10A.1F1 | - |
| **G10A (10 TgShSp1 oocysts)** |  |  | 10A.1F2 | 1:64 |
|  | **10A.2** | a | 10A.2F1 | 1:100 |
|  |  |  | 10A.2F2 | 1:100 |
|  | **10A.3** | a | 10A.3F1 | 1:200 |
|  |  |  | 10A.3F2 | - |
|  |  |  | 10A.3F3 | 1:100 |
|  | **10A.4** | a | 10A.4F1 | -^ |
|  | **10A.5** | a | 10A.4F2 | 1:16^ |
|  |  |  | 10A.5F1 | 1:200 |
|  |  |  | 10A.5F2 | NA* |
|  | **10A.6** | a | 10A.6F1 | 1:200 |
|  |  |  | 10A.6F2 | 1:100 |
|  |  |  | 10A.6F3 | 1:800 |
|  |  |  | 10A.6F4 | - |
| **G10B (10 TgME49 oocysts)** | **10B.1** | 11 | 10B.1F1 | - |
|  |  |  | 10B.1F2 | - |
|  | **10B.2** | a | 10B.2F1 | 1:50 |
|  | **10B.3** | a | 10B.3F1 | - |
|  |  |  | 10B.3F2 | -^ |
|  | **10B.4** | a | 10B.4F1 | NA* |
|  | **10B.5** | a | 10B.5F1 | 1:50 |

^a^ Day post-challenge when foetal death was detected by ultrasonography. The remaining ewes (*a*) delivered stillbirths/live lambs

^ thoracic or abdominal fluids were analysed in stillborn lambs of which it was unable to obtain precolostral sera. ^*^ suckling before serum sampling. dpi: days post-infection; FL: foetal liquid; NA: not available.
